# Mechanism of Metabolic Response to Hepatectomy by Integrated Analysis of Gut Microbiota, Metabolomics, and Proteomics

**DOI:** 10.1128/spectrum.02067-22

**Published:** 2023-04-10

**Authors:** Ruoxuan He, Shuang Gao, Hua Yao, Zixuan Zhao, Jinjin Tong, Hua Zhang

**Affiliations:** a Animal Science and Technology College, Beijing University of Agriculture, Beijing, People’s Republic of China; Wayne State University

**Keywords:** hepatectomy, gut microbiology, metabolomics, proteomics

## Abstract

Hepatectomy is a common clinical procedure for the treatment of many liver diseases, and the successful recovery of a patient’s liver metabolism and function after surgery is crucial for a good prognosis. The objective of this study was to elucidate the metabolic response to hepatectomy using high-throughput sequencing analysis of 16S rRNA gene, metabolomics, and proteomics data. Fecal and serum samples from beagle dogs were collected on day 0 (LH0), day 7 (LH7), and day 28 (LH28) after laparoscopic partial hepatectomy. Liver tissue samples were taken on LH0 and LH7. Dysbiosis in the fecal microbiota was explored, and host-microbiome interactions based on global metabolic and protein profiles and inflammatory processes were determined. Results showed that the relative abundance of *Allobaculum* and *Turicibacter* was decreased and that of Escherichia-*Shigella* was increased after hepatectomy (*P* < 0.05); the phenylalanine, tyrosine, and tryptophan biosynthetic pathway, along with the phenylalanine and aminoacyl-tRNA biosynthetic pathway, was significantly associated with liver injury. The serum metabolites l-phenylalanine and l-arginine were useful as biomarkers, and the fecal metabolite l-threonine was a signature target monitor for liver recovery. The proteomics profile revealed 412 significantly different proteins and further highlighted two key signaling pathways (mitogen-activated protein kinase [MAPK] and peroxisome proliferator-activated receptor [PPAR]) involved in the response to liver injury. We systematically explored the metabolic mechanism of liver injury and recovery, providing new insights into effective ways to promote recovery after hepatectomy and improve liver function and long-term survival. These fundamental studies on hepatectomy will provide the basis for future advances in treatment and recovery from common liver diseases.

**IMPORTANCE** As the largest parenchymal organ, the liver is a target for bacterial and viral infections, nonalcoholic fatty liver disease (NAFLD), cirrhosis, cancer, and many other diseases, constituting a serious worldwide problem. The treatment for many of these diseases involves hepatectomy. Here, we show that aberrant inflammatory processes after hepatectomy of the liver as reflected in the association between liver metabolism and gut microbiota create a grave risk. This study investigated the mechanisms of gut microbiota and host metabolism involved in liver injury and recovery after hepatectomy, using proteomics to reveal the mechanisms of postoperative liver injury and a comprehensive multi-omics approach to identify changes in metabolism after hepatectomy.

## INTRODUCTION

Liver disease and its complications are one of the leading causes of illness and death worldwide. In past decades, hepatectomy has been established as a safe primary treatment for various benign and malignant diseases of the liver (1). Until now, accumulated advances in research have led to significant achievements in understanding the characteristics and progress of hepatectomy, as a result of tumors, cirrhosis, and liver failure. Sahay et al. (2) established an acute liver failure model in rats by performing 70% partial hepatectomy (PHx) and consequently evaluated the therapeutic potential of cells in bridging the gap between acute liver failure and liver transplantation. The PHx rat model has been widely used since then to study the involvement of regeneration in the recovery mechanism from acute liver injury (3). Hepatic resection is characterized by a catabolic state, in which the body’s metabolism as reflected in serum and urine is significantly altered (4). These injury-induced metabolic changes may adversely affect liver regeneration as well as functional recovery, and even the functions of other organs. Although several studies have focused on the central role of the liver in metabolic regulation, the mechanisms of interaction of inflammation, microbiota, hepatic metabolism, and liver regeneration after hepatectomy are poorly understood. It is important, therefore, to explore the mechanisms of metabolic changes in response to hepatectomy, with the goal of developing novel therapeutic regimens with better efficacy and safety for reducing liver injury and disease.

The intestinal microbiota have widely been considered to play a key role in preserving liver homeostasis. Current research has focused on the integrative functional and physiological connections between the liver and the intestine, which are known as the hepatic-intestinal axis (5). Preclinical studies showed that intestinal microbiota, acting through the gut-liver axis, could significantly reduce liver injury and enhance regeneration through their influence on proliferation and metabolism (6). Other studies confirmed the correlation between changes in the composition and activity of the gut microbial population and the progression of liver diseases such as alcoholic and nonalcoholic fatty liver disease (NAFLD), steatohepatitis, cirrhosis, and hepatocellular carcinoma (7). Conversely, the liver also exerts an influence over intestinal microbial communities via secretion of bile acids and IgA antibodies (8). Interestingly, some studies have found that liver ischemia-reperfusion injury during surgery can cause changes in intestinal communities, resulting in decreased abundance of *Lactobacillus*, *Bifidobacterium*, and *Bacteroides* and increased abundance of *Enterococcus* and *Enterobacteriaceae* (9). *Lactobacillus*, *Bifidobacterium*, and Bacillus mimicus have been found to be closely associated with liver injury and regeneration. Specifically, Lactobacillus rhamnosus was found to correct intestinal dysbiosis and reduce liver fat accumulation and cellular inflammatory responses in mice with alcoholic liver injury (10). Collectively, gut microbes play a significant role in the etiology of hepatic diseases and the processes of liver injury and repair. However, a comprehensive picture of the underlying microbial interactions and the associated posthepatectomy mechanisms remains elusive.

With the development of advanced bioinformatics analysis of the microbiome through proteomics and metabolomics, the functional activity of the gut microbial community and the mechanisms involved in metabolic change can now be more fully understood (11). Thus, this holistic, integrative, multi-omics approach can give us a clearer picture of how changes in the level of metabolites after hepatectomy can be applied in diagnosing liver injury and monitoring the recovery progress. In the present study, we integrated data on the microbiota, proteome, and metabolome to clarify the underlying mechanisms of hepatectomy and establish a cross-sectional framework that will provide a useful reference for future exploration of new therapies for liver disease.

## RESULTS

### Partial hepatectomy modeling.

In this study, the four-trocar method (two 5-mm trocars and two 10-mm trocars) was used to successfully establish a canine model of laparoscopic left hemihepatectomy in eight dogs. All the dogs recovered from anesthesia within 30 min of the completion of the surgery, and no major intraoperative or postoperative complications occurred. The total length of the incision was 6.7 ± 0.54 cm, the average operative time was 102.53 ± 9.07 min, and the estimated blood loss was 32.10 ± 6.43 mL (*P* < 0.05). No complications occurred during the entire experiment, and none of the dogs manifested weight loss by 1 month after surgery.

### Changes in liver function and inflammatory indices.

Compared to before surgery (LH0), the serum aspartate transferase (AST) and alanine transaminase (ALT) levels were increased on LH1, LH3, and LH7 (*P* < 0.01) and then gradually returned to preoperative levels by LH28 (Table 1). The serum tumor necrosis factor alpha (TNF-α) concentration showed similar changes, while serum interleukin-1β (IL-1β) and IL-6 concentrations showed significant increases on LH1, LH3, and LH7 (*P* < 0.01) and then decreased on LH14 and LH28 (Table 1). In summary, serum levels of TNF-α, IL-1β, and IL-6 were increased significantly at LH1, and the difference was extremely significant compared with LH0 (*P* < 0.01); levels continued to increase and reached their highest point at LH3. In contrast, serum levels of the anti-inflammatory factors IL-4 and IL-10 were significantly lower on LH1, LH3, and LH7 (*P* < 0.01) and then tended to increase, gradually returning to preoperative levels by LH28.

**TABLE 1:** Serum enzymes of liver function and inflammatory cytokines^*a*^

Enzyme	Preop	LH1	LH3	LH7	LH14	LH28
AST (IU/L)	19.6 ± 2.31	260.61 ± 300.88**	49.49 ± 5.78**	27.59 ± 2.36	23.54 ± 1.85	19.42 ± 1.95
ALT (U/L)	28.28 ± 8.42	294.83 ± 730.84**	210.31 ± 410.47**	41.61 ± 26.29	30.72 ± 15.15	31.99 ± 9.62
TNF-α (pg/mL)	42.54 ± 4.40	61.69 ± 5.62**	71.14 ± 5.32**	51.78 ± 2.33	46.03 ± 3.74	40.32 ± 3.79
IL-1β (pg/mL)	27.01 ± 1.30	34.32 ± 2.42**	45.36 ± 2.55**	31.81 ± 1.44**	28.30 ± 2.80	29.11 ± 1.41
IL-6 (pg/mL)	87.33 ± 4.51	131.58 ± 1.30**	155.29 ± 4.34**	121.25 ± 7.13**	105.38 ± 4.56*	85.63 ± 2.47
IL-4 (pg/mL)	14.36 ± 1.17	10.28 ± 0.17**	7.82 ± 0.28**	11.94 ± 0.62**	14.05 ± 0.39*	14.58 ± 2.09
IL-10 (pg/mL)	22.56 ± 1.24	15.50 ± 0.46**	11.66 ± 0.67**	16.74 ± 0.55**	18.90 ± 0.38**	23.27 ± 1.74

a Data shown as mean ± standard deviation, *n* = 8. Preop, preoperation; POD, postoperative day. Compared with preoperation, *, *P* < 0.05; **, *P* < 0.01. AST, aspartate transferase; ALT, alanine transaminase; TNF, tumor necrosis factor; IL, interleukin.

### Gut microbiota altered after hepatectomy.

**FIG 1:**
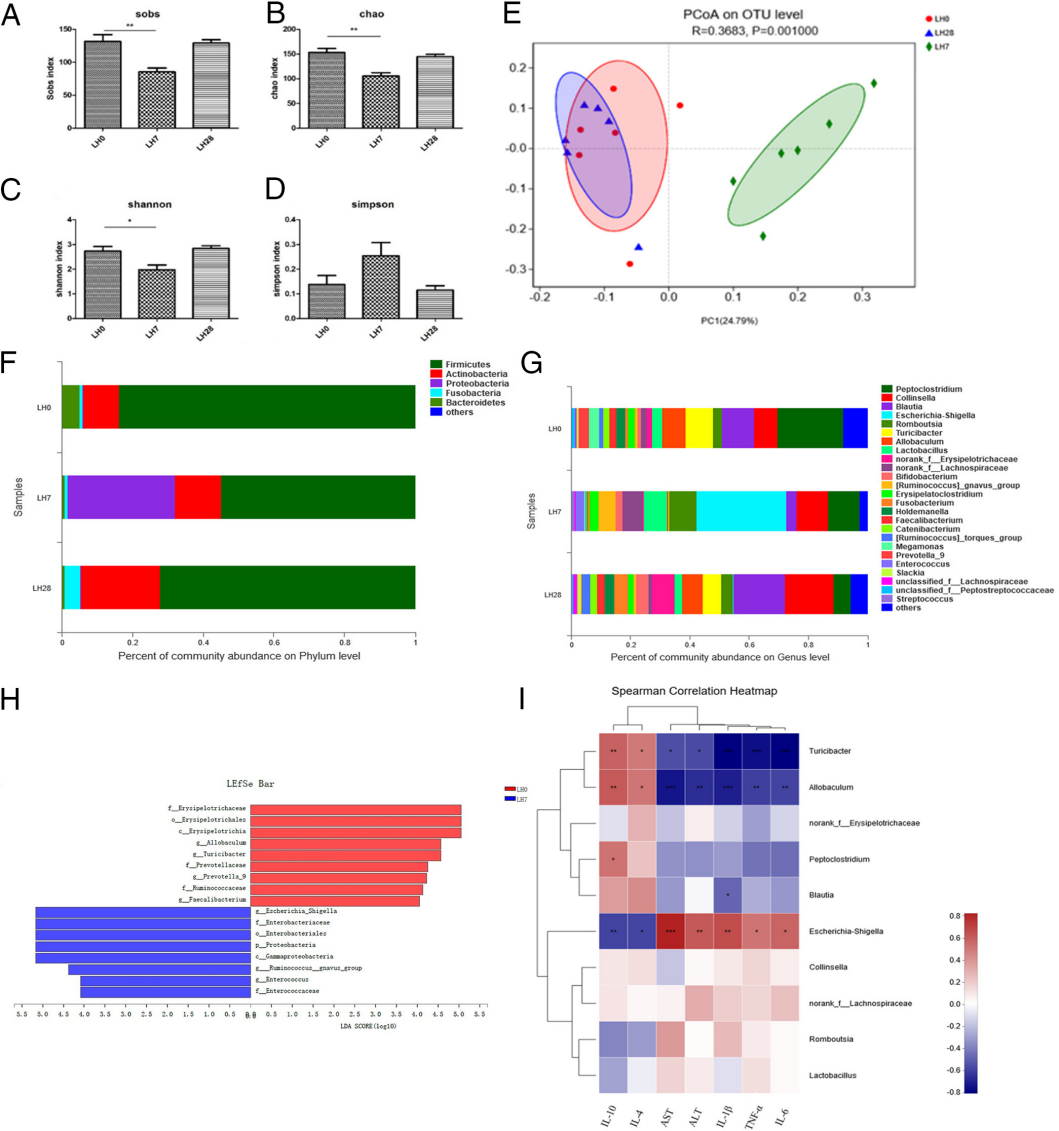
(A to D) Alpha diversity indices at different time points before and after surgery. (A) Sobs index; (B) Chao index; (C) Shannon index; (D) Simpson index. LH0, the morning before surgery; LH7, day 7 after surgery; LH28, day 28 after surgery. (E) Principal-coordinate analysis (PCoA) of microbial communities. Circle, rhombus, and triangle represent the gut microbiota of the morning before surgery (LH0), day 7 after surgery (LH7), and day 28 after surgery (LH28), respectively. Distances between symbols on the ordination plot reflect relative dissimilarities in community composition. (F) Relative abundances of different bacteria at the phylum level of each group. (G) Relative abundances of different bacteria at the genus level of each group. (H) LEfSe identified the differences in most abundant taxa between LH0 and LH7. The enriched taxa on LH0 are indicated with a positive LDA score (red), while the taxa enriched on LH7 have a negative score (blue). Only the taxa having a *P* value of <0.01 and an LDA score of >4.0 are shown. (I) Heatmap of the Spearman correlations between the top 10 most abundant genera and clinical parameters such as inflammatory cytokines TNF-α, IL-1β, IL-4, IL-6, and IL-10 and liver function indices ALT and AST. *, 0.01 < *P* ≤ 0.05; **, 0.001 < *P* ≤ 0.01; ***, *P* ≤ 0.001.

To investigate the changes in diversity of gut microbiota in response to hepatectomy, we first estimated alpha diversity in fecal samples by calculating four important parameters (Sobs, Chao, Shannon, and Simpson). Compared to LH0 (*n* = 8), the Sobs (*P* < 0.05) and Chao (*P* < 0.01) indices were significantly decreased on LH7 (Fig. 1A and B), indicating a decrease in the level of intestinal microbiota richness after hepatectomy. It is worth noting that the higher Shannon index and the lower Simpson index indicated high species diversity; the Shannon index (Fig. 1C) was decreased and the Simpson index (Fig. 1D) was increased on LH7, indicating a decrease in intestinal microbiota diversity after hepatectomy. However, on LH28, all four indices show a gradual recovery back to LH0 levels. The changes in the above indices indicated that the abundance and diversity of intestinal microbiota in beagle dogs declined up to LH7 and recovered by LH28 (Fig. 1A to D). We used principal-coordinate analysis (PCoA) based on the Bray-Curtis metric to examine the community structures of the gut microbiota of the dogs before and after hepatectomy (Fig. 1E, *R* = 0.3683, *P* = 0.001). Bacterial communities at LH0 and LH28 were clearly separated from LH7 along principal-coordinate axis 1 (PC1) (*P* = 0.001), indicating that the composition of the gut microbiota was different (Fig. 1E). This finding suggests that hepatectomy had significant effects on the microbiota composition.

We further investigated the specific changes in the microbiota after hepatectomy by assessing the relative abundance of taxa (Fig. 1F). For differential abundance comparison analysis at the phylum level, the relative abundance of *Proteobacteria* was significantly increased on LH7 (*P* < 0.01) relative to LH0 (Table 2). At the genus level, the bacterial taxa that displayed differential abundance on LH7 and LH28 relative to LH0 are shown in Fig. 1G. The relative abundance of *Peptoclostridium* decreased after hepatectomy, and the difference between LH7 and LH0 was very significant (*P* < 0.01), while the difference between LH28 and LH0 was significant (*P* < 0.05). Compared to LH0, the most common opportunistic pathogens in the gut (Escherichia*-Shigella*) were overrepresented on LH7 (*P* < 0.01); conversely, compared to LH0, the relative abundance of *Turicibacter* and *Allobaculum* decreased on LH7 (*P* < 0.05) (Table 3).

**TABLE 2:** Relative abundance and diversity test of the top five gut bacterial groups at the phylum level before and after surgery^*a*^

Phylum	LH0 (%)	LH7 (%)	LH28 (%)	*P* value
*Firmicutes*	83.53 ± 14.88	54.56 ± 29.32	71.52 ± 15.97	0.31
*Actinobacteria*	10.09 ± 6.99	13.27 ± 9.44	22.47 ± 11.27	0.31
*Proteobacteria*	0.11 ± 0.06 b	30.58 ± 25.39 A	0.19 ± 0.31 b	0.03
*Fusobacteria*	0.99 ± 1.92	0.74 ± 1.48	4.91 ± 6.91	0.31
*Bacteroidetes*	5.25 ± 11.66	0.81 ± 0.84	0.81 ± 0.32	0.66

a Data are shown as mean ± standard deviation, *n* = 6. The values of the same data at different time points were compared from high to low. If two values have the same following letters (except for case), no significant difference was found (*P* > 0.05). If the letters are not the same, there is a significant difference. If the letter is uppercase, there is a significant difference (*P* < 0.05), and if the letter is lowercase, there is a very significant difference (*P* < 0.01).

**TABLE 3:** Relative abundance and diversity test of the top 10 gut bacterial groups at the genus and family level before and after surgery^*a*^

Taxon level	LH0 (%)	LH7 (%)	LH28 (%)	*P* value
Genus				
*Peptoclostridium*	22.44 ± 17.13 A	4.34 ± 3.71 b	6.25 ± 3.38 B	0.007
*Collinsella*	10.10 ± 7.21	8.20 ± 4.84	16.86 ± 10.39	0.29
*Blautia*	11.45 ± 7.61	3.86 ± 2.92	10.25 ± 7.34	0.08
Escherichia-*Shigella*	0.05 ± 0.05 b	37.13 ± 22.21 A	0.12 ± 0.18 b	0.007
*Romboutsia* (%)	2.83 ± 1.81	10.46 ± 9.36	5.07 ± 2.95	0.26
*Turicibacter*	9.11 ± 6.87 A	0.17 ± 0.19 B	6.57 ± 5.76 AB	0.007
*Allobaculum*	6.25 ± 2.95 A	0.41 ± 0.87 B	7.26 ± 5.63 A	0.007
*Lactobacillus*	0.57 ± 1.03	10.58 ± 12.53	4.68 ± 6.50	0.11
Family				
*Erysipelotrichaceae*, no rank	0.59 ± 0.85 b	0.40 ± 0.39 b	6.99 ± 2.34 A	0.007
*Lachnospiraceae*, no rank	1.02 ± 0.69	2.98 ± 3.66	1.20 ± 0.49	0.70

a Data are shown as mean ± standard deviation, *n* = 6. Significance is shown similarly to Table 2.

Next, we applied linear discriminant analysis (LDA) coupled with effect size (LEfSe) to explore the significance of changes in, and relative enrichment of, bacterial communities on LH0 and LH7. The LEfSe analysis revealed a significant increase in Escherichia-*Shigella*, *Ruminococcus*, and *Enterococcus* and a substantial reduction in *Allobaculum*, *Turicibacter*, *Prevotella*, and *Faecalibacterium* on LH7 relative to LH0 (Fig. 1H).

### Associations between clinical parameters and gut microbiota.

We investigated the correlation between the relative abundance of the top 10 most abundant genera that showed significant differences in LH7 and the clinical parameters (Fig. 1I). *Allobaculum* and *Turicibacter* were significantly negatively correlated with AST, ALT, TNF-α, IL-1β, and IL-6, and positively correlated with IL-10 and IL-4, whereas Escherichia-*Shigella* was the opposite. These findings suggest that the changes in liver function and inflammatory response caused by hepatectomy were closely related to the relative abundance of *Allobaculum*, *Turicibacter*, and Escherichia-*Shigella*. When the liver is damaged and an inflammatory response occurs, the relative abundance of *Allobaculum* and *Turicibacter* is decreased, while Escherichia-*Shigella* proliferation is increased (Fig. 1I).

### Alteration in metabolomics after hepatectomy.

**FIG 2:**
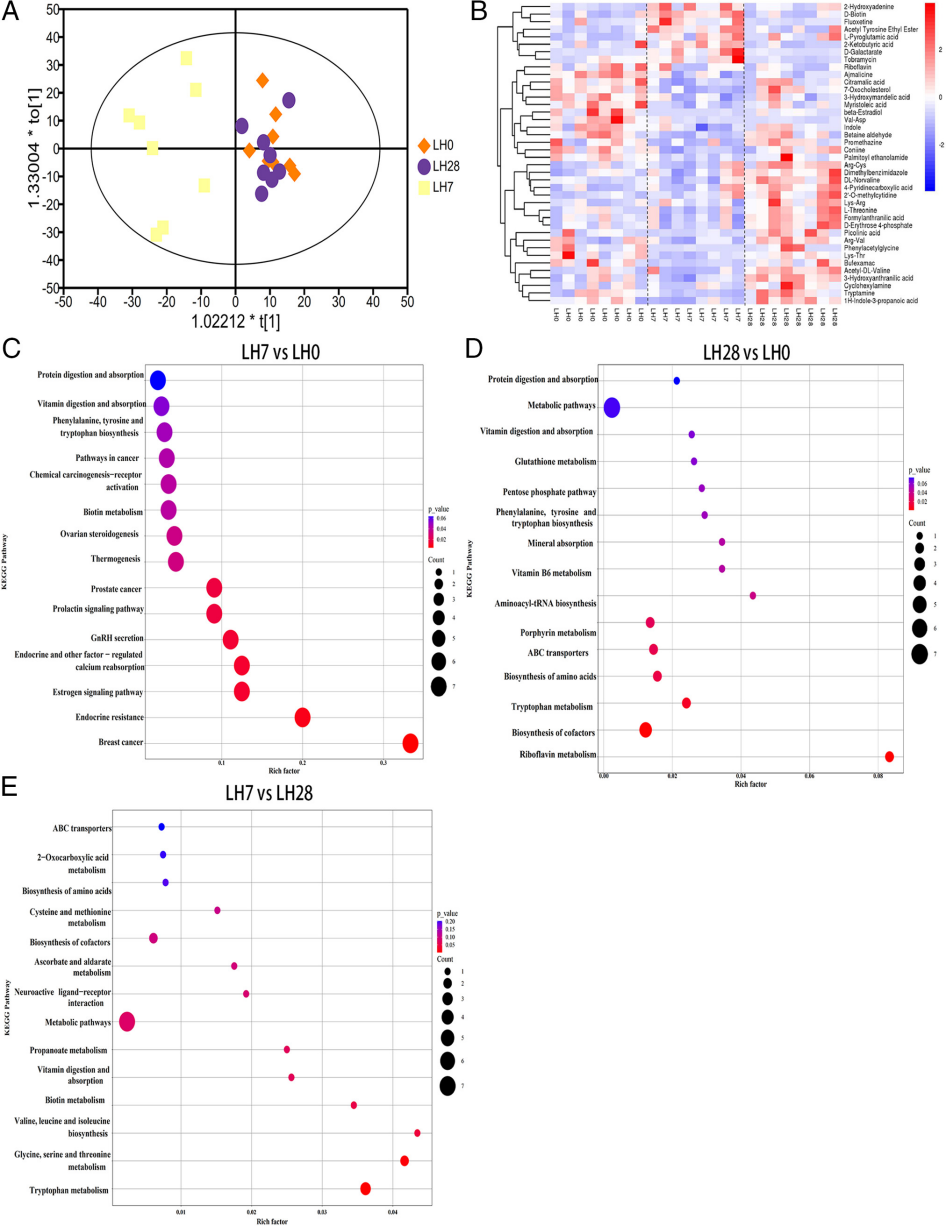
Fecal metabolomics at different time points before and after surgery. (A) Scatterplot of OPLS-DA model for LH0, LH7, and LH28. (B) Heatmap showing differential fecal metabolite changes between the three groups. (C to E) Pathway enrichment analysis performed using the significantly different fecal metabolites between the three groups: LH0 versus LH7 (C), LH0 versus LH28 (D), and LH7 versus LH28 (E).

Considering the interplay between the gut microbiome and host metabolism, we performed untargeted metabolomics on fecal samples. An orthogonal correction partial least-squares discriminant analysis (OPLS-DA) revealed that the metabolic compositions of LH0 and LH28 were clearly different from that of LH7, indicating that the composition of fecal metabolites was significantly altered during the 7 days after hepatectomy. However, the distribution of fecal samples on LH28 and LH0 was more aggregated together, indicating that the altered fecal metabolome gradually recovered to preoperative levels during the 28 days postoperatively (Fig. 2A). Next, we identified the first 15 significantly different metabolites based on the OPLS-DA model with variable importance in projection (VIP) of >1 and *P* of <0.05 (two-tailed Student’s *t* test). There were 11 different metabolites downregulated and four metabolites upregulated on LH7 compared to LH0 (see Table S1 in the supplemental material). The heatmap of hierarchical clustering (Fig. 2B) shows that at LH7, the upregulated metabolites include d-biotin and d-galactarate, and the downregulated metabolites include phenylacetylglycine, involved in phenylalanine, tyrosine, and tryptophan biosynthesis, and betaine aldehyde, involved in glycine, serine, and threonine metabolism. Compared to LH0, metabolites such as l-threonine were upregulated and riboflavin was downregulated on LH28 (Fig. 2B and Table S2). As described above, we identified the significantly different metabolites between preoperative and postoperative time points and determined the major enrichment pathways for these differentially produced metabolites based on KEGG and PubChem online databases. Compared to LH0, the major enrichment pathways for the differential metabolites on LH7 included phenylalanine, tyrosine, and tryptophan biosynthesis and biotin metabolism (Fig. 2C). On LH28, the enrichment pathways included aminoacyl-tRNA biosynthesis and riboflavin metabolism, among others (Fig. 2D). In the comparison between LH7 and LH28, pathway enrichment analysis revealed that the main pathways included glycine, serine, and threonine metabolism, among others (Fig. 2E).

**FIG 3:**
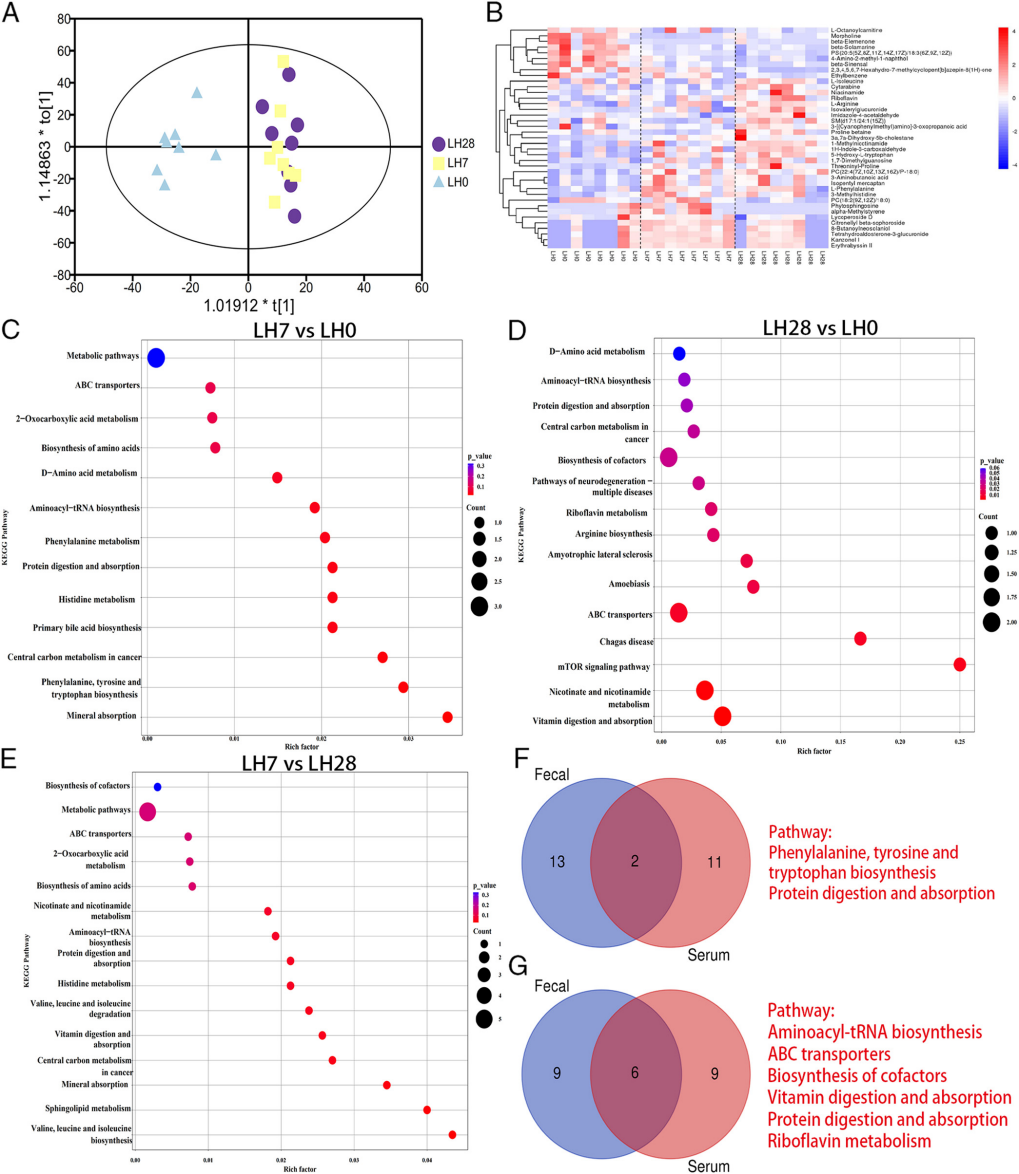
Posthepatectomy serum metabolome. (A) Comparison of serum metabolome between LH0 and LH7 and between LH0 and LH28 visualized using OPLS-DA. (B) Heatmap of the differential metabolites identified in serum samples. (C to E) Pathway enrichment analysis performed using the significantly different serum metabolites between the three groups: LH0 versus LH7 (C), LH0 versus LH28 (D), and LH7 versus LH28 (E). (F and G) Venn diagram illustrating the common metabolic pathways of fecal and serum samples (F: LH0 versus LH7; G: LH0 versus LH28).

To comprehensively analyze the mechanism of metabolic changes in liver injury and recovery, we also performed serum metabolomics analyses. The metabolites in serum samples were identified by ultrahigh-pressure liquid chromatography (UHPLC)–quadrupole time of flight mass spectrometry (QTOFMS), and the data were normalized to a defined OPLS-DA model with a cumulative R2(Y) of 0.972 and a Q2 value of 0.917, showing a trend of separation in LH7 and LH28 compared to LH0 (Fig. 3A). We further screened for differential metabolites by a VIP of >1 and a *t* test, with a *P* value of <0.05 as the selection criterion in the OPLS-DA model. The heatmap shows that, compared to LH0, the upregulated metabolites in LH7 include lipids and lipid-like molecules such as 3a,7a-dihydroxy-5b-cholestane and l-phenylalanine, which is involved in phenylalanine, tyrosine, and tryptophan biosynthesis, phenylalanine metabolism, and aminoacyl-tRNA biosynthesis (Fig. 3B and Table S3). On LH28, the main differential metabolites were l-arginine and riboflavin, both of which were upregulated (Fig. 3B and Table S4). However, the downregulation of differential serum metabolites that occurred on LH28 was not significant. To further clarify the metabolic pathways and functions during regeneration, a comprehensive analysis of the significant differential metabolites identified was performed using PubChem and KEGG online databases. The results showed that, compared to LH0, the differential metabolites were mainly enriched in phenylalanine, tyrosine, and tryptophan biosynthesis, phenylalanine metabolism, and primary bile acid biosynthesis on LH7 (Fig. 3C). On LH28, they were mainly enriched in aminoacyl-tRNA biosynthesis, riboflavin metabolism, and nicotinate and nicotinamide metabolism (Fig. 3D).

Summarizing the metabolomic results, compared to the preoperative period, the represented pathways involved in the biosynthesis and metabolism of phenylalanine were identified in both fecal and serum metabolomics on LH7 (Fig. 3E). The enriched pathway for aminoacyl-tRNA biosynthesis was present for significantly different fecal and serum metabolites on LH28 (Fig. 3G). This finding reinforces the significant effects of both metabolic pathways on metabolic changes after hepatectomy, and we hypothesized that the fecal metabolite phenylacetylglycine and the serum metabolite l-phenylalanine, which are part of the phenylalanine biosynthesis and metabolism pathway, could be used as biomarkers for liver injury. The fecal metabolite l-threonine and the serum metabolite l-arginine from the aminoacyl-tRNA biosynthetic pathway could be used as key target metabolites for the liver recovery process.

### Associations between the metabolome, clinical parameters, and gut microbiome.

**FIG 4:**
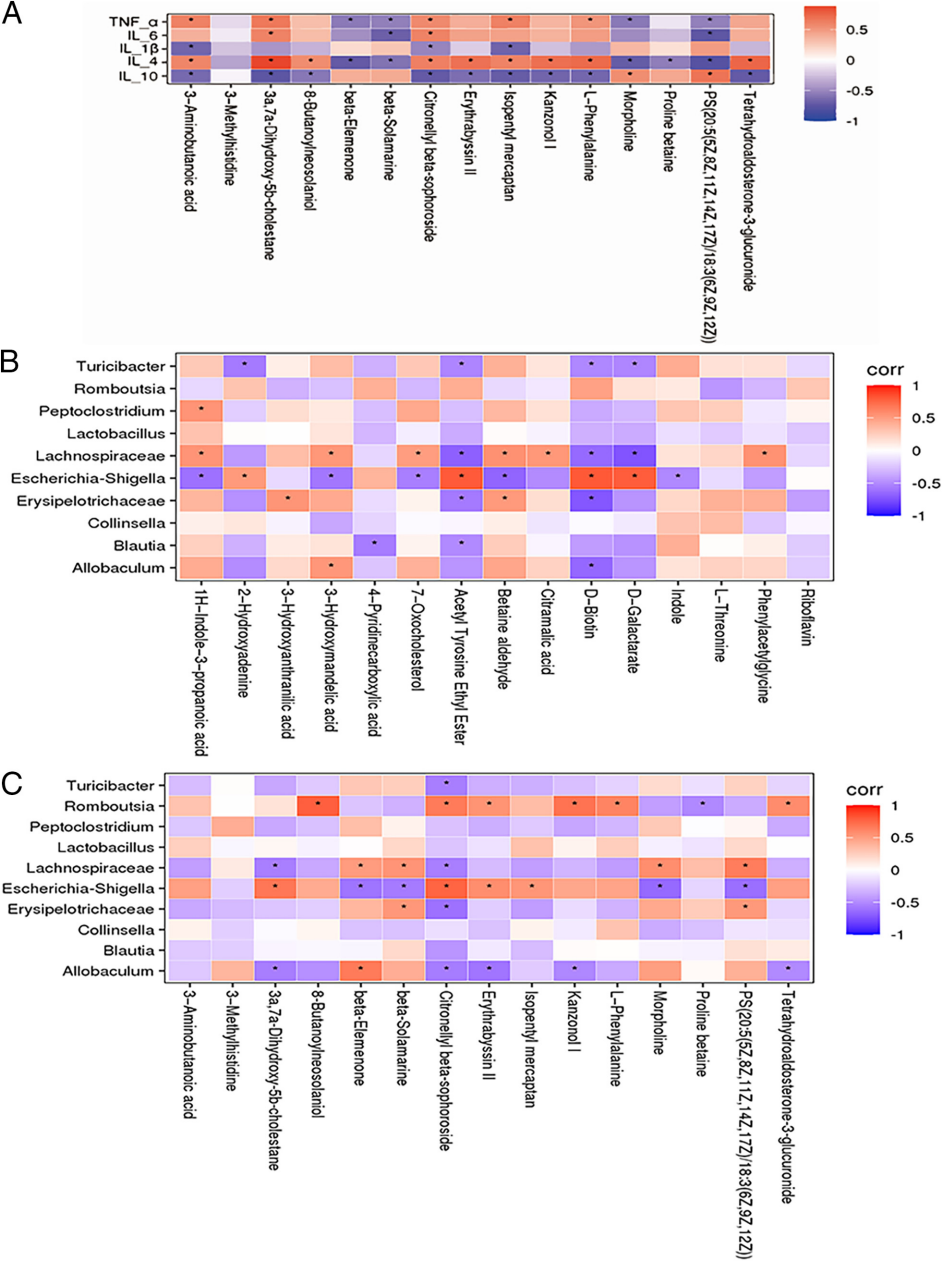
Comparing LH0 with LH7, the top 10 most abundant genera in the gut microbiome and the top 15 fecal and serum differential metabolites with significant differences based on previous analyses, as well as the five main inflammatory indicators, were selected for further correlation analysis. (A) The heatmap shows the association between the five significantly different clinical serum variables and the serum metabolites with significant differences (*, *P* ≤ 0.05). (B and C) The heatmap depicts the association between the most abundant genera in the gut microbiome and the metabolites in the fecal samples (B) and serum samples (C) (*, *P* ≤ 0.05).

We assessed the association between the serum level of five significantly different clinical parameters, TNF-α, IL-6, IL-4, IL-1β, and IL-10, and the serum level of metabolites (Fig. 4A and Table S5). We found that the serum metabolite l-phenylalanine, which was increased at LH7 after hepatectomy, and 3a,7a-dihydroxy-5b-cholestane, which is involved in primary bile acid biosynthesis, were positively correlated with the proinflammatory factors TNF-α, IL-6, and IL-1β but negatively correlated with anti-inflammatory factors IL-4 and IL-10, compared to LH0 (*P* < 0.05). The differences in levels of these serum metabolites can be used to assess the degree of liver damage and to further explore their role in liver recovery.

Since the physiological impact of the gut microbiota on the host is often mediated by a complex host-microbe metabolic axis, we next analyzed the associations between the abundance of specific bacterial genera and metabolites related to liver injury. In the gut microbiota, the abundance of the potentially pathogenic Escherichia-*Shigella* group was negatively correlated with downregulated fecal metabolites such as betaine aldehyde and phenylacetylglycine but positively correlated with the upregulated fecal metabolites d-galactarate and d-biotin and the serum metabolites l-phenylalanine and 3a,7a-dihydroxy-5b-cholestane. Notably, these upregulated metabolites were negatively correlated (*P* < 0.05), or less correlated, with the abundance of *Allobaculum* and *Turicibacter* species that underwent downregulation due to liver injury (Fig. 4B and C and Tables S6 and S7). In addition to the previously characterized liver injury-related species, these metabolites also exhibited a close association with liver injury inflammatory factors. This further supports the hypothesis that changes in the metabolic mechanisms of liver injury are orchestrated by a complex, interacting host-microbe metabolic axis.

### Influence of hepatectomy on proteomics.

**FIG 5:**
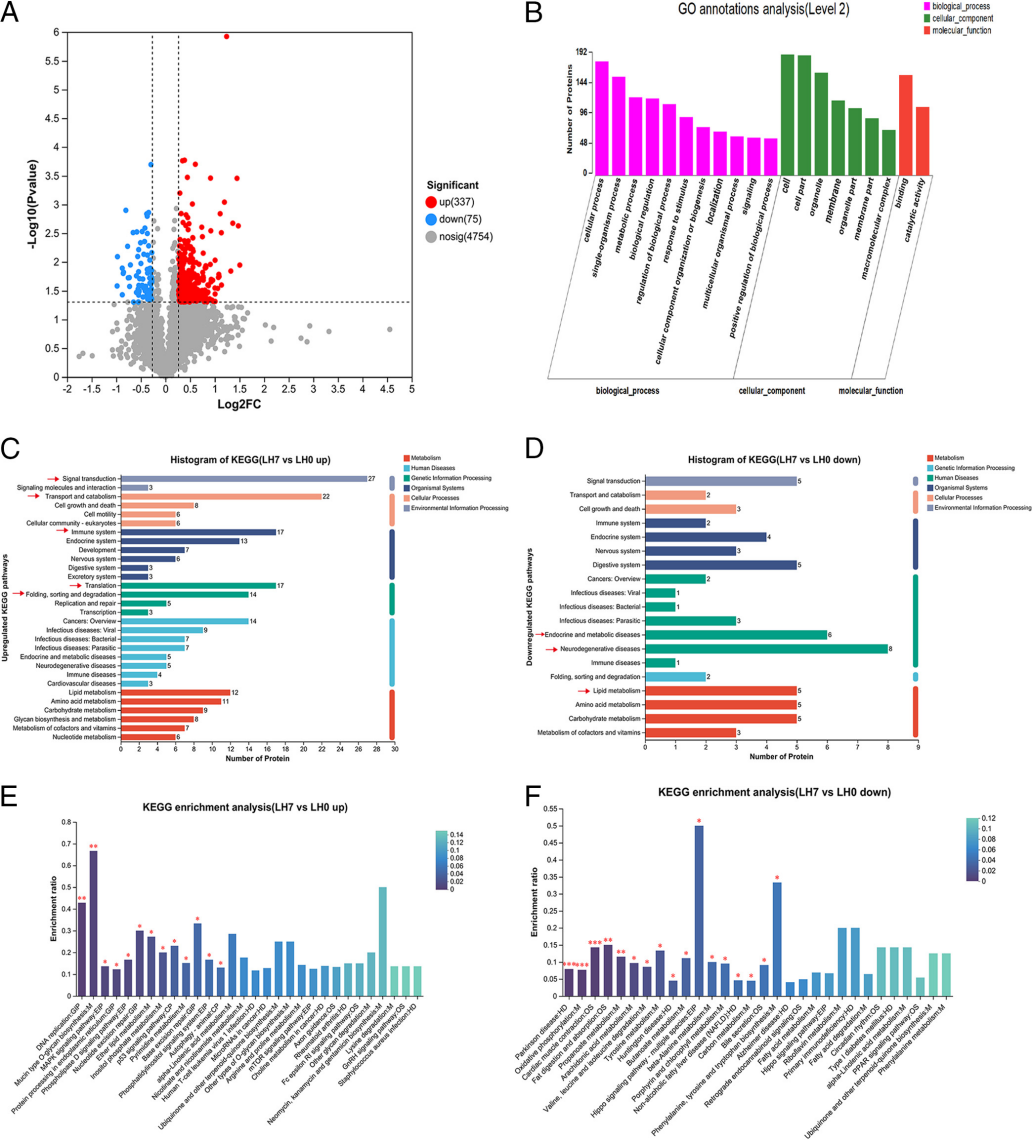
Proteomics analysis and comparison between LH0 and LH7. (A) Changes in the hepatic proteome. Alterations in hepatic proteins were demonstrated by volcano plots showing log_2_-transformed changes in protein abundance (versus control) on the *x* axis with log_10_-transformed *P* values on the *y* axis (*P* < 0.05). Red denotes significantly upregulated proteins and blue denotes significantly downregulated proteins meeting the predefined fold change (FC of >1.2 or <0.83). (B) GO enrichment analysis of differential proteins between LH0 and LH7 (*P* < 0.05). (C and D) KEGG pathway analysis of differential proteins between LH0 and LH7 (*P* < 0.05). (C) Upregulated differential proteins. (D) Downregulated differential proteins. (E and F) KEGG enrichment analysis of differential protein between LH0 and LH7. Column color gradients indicate the significance of enrichment, with darker colors by default representing more significant enrichment for that KEGG term, where *P* < 0.001 is marked as ***, *P* < 0.01 is marked as **, and *P* < 0.05 is marked as *. (E) Upregulated differential proteins. (F) Downregulated differential proteins.

To further elucidate the mechanism of the metabolic changes brought about by hepatectomy, we performed proteomic analysis on liver tissue from LH0 and LH7. Of the 5,166 proteins that could be quantified in this experiment, a total of 412 proteins were significantly differentially expressed between LH0 and LH7; the number of upregulated proteins was 337, and the number of downregulated proteins was 75 (fold change [FC] of >1.2 or <0.83 and *P* value of <0.05) (Fig. 5A and Table S8). The differentially expressed proteins were analyzed by searching the Gene Ontology (GO) database to determine their participation in biological processes (BPs) or molecular functions (MFs) and the type of cellular component (CC) with which they were associated. The BP category analysis revealed that the proteins were mainly involved in cellular processes, single-organism processes, and metabolic processes (Fig. 5B). The classification according to CC showed that the proteins were mainly related to cells, cell parts, and specific organelles. With regard to MF, the differentially expressed proteins were associated with protein binding and catalytic activity (Fig. 5B).

The Kyoto Encyclopedia of Genes and Genomes (KEGG) enrichment analysis was carried out to determine which metabolic pathways the differentially expressed proteins were part of. Among the upregulated differential proteins, the top five authoritative KEGG pathways (*P* < 0.05) were signal transduction, transport and catabolism, translation, immune system, and the folding, sorting and degradation (Fig. 5C). The metabolic pathways involved with the higher number of downregulated differential proteins mainly included neurodegenerative diseases, lipid metabolism, and endocrine and metabolic diseases (Fig. 5D). Further analysis of signaling pathways significantly enriched in KEGG function using Fisher’s exact test, with all proteins as background (*P* < 0.05), revealed that the upregulated differential proteins were mainly involved in mucin type O-glycan biosynthesis, DNA replication, and mitogen-activated protein kinase (MAPK) signaling, among others (Fig. 5E). The downregulated differential proteins were mainly involved in oxidative phosphorylation, fat digestion and absorption, peroxisome proliferator-activated receptor (PPAR) signaling, phenylalanine, tyrosine, and tryptophan biosynthesis, and phenylalanine metabolism (Fig. 5F). Notably, the proteomics analysis confirmed the metabolomics data in that the phenylalanine, tyrosine, and tryptophan biosynthesis and phenylalanine metabolic pathways were significantly related to liver injury.

## DISCUSSION

In this study, by integrating fecal microbiota, metabolomics, and proteomics data, we investigated the mechanisms of metabolic changes in response to hepatectomy. The contribution of microbiota and metabolites to liver metabolism and the signaling pathways involved in the mechanisms of liver injury development were revealed.

In the laparoscopic approach, the venous oozing can be effectively manipulated by elevation of the pneumoperitoneal pressure (12). This is particularly important for patients with cirrhosis where venous oozing secondary to portal hypertension is a common phenomenon. It has been shown that laparoscopic hepatectomy performed within the range of 10 to 14 mm Hg was safe (13–16). A pneumoperitoneal pressure of 1.33 kPa was found to be optimal for laparoscopic surgery in dogs; too high a pneumoperitoneal pressure can lead to severe changes in circulatory indicators (17). Therefore, we recommend a pneumoperitoneal pressure setting of 8 to 10 mm Hg.

Our study showed that the specificity of gut microbial response to hepatectomy was confirmed by 16S rRNA gene high-throughput sequencing. At the genus level, the abundance of *Allobaculum* and *Turicibacter* was significantly decreased, while Escherichia-*Shigella* increased after hepatectomy, accompanied by an increase in inflammatory factors. Thus, we hypothesized that *Allobaculum* and *Turicibacter* were involved in boosting the production of anti-inflammatory factors in the body, protecting the intestinal barrier, and reducing liver damage. Previous studies reported that *Allobaculum* had a strong fermentative capacity and released short-chain fatty acids (SCFAs) during the degradation of dietary fiber. SCFAs are considered potential mediators of intestinal immune function, including inhibiting the production of proinflammatory factors and maintaining intestinal barrier function (5). Nonalcoholic fatty liver disease also causes a decrease in *Turicibacter* abundance, aggravating liver inflammation and decreasing intestinal function and serum metabolic index (18). Consistent with these results, we found that the relative abundances of *Allobaculum* and *Turicibacter* were significantly decreased after hepatectomy, suggesting that lobectomy had a significant negative effect on the microbiota.

Escherichia*-Shigella* is an opportunistic pathogen, and an increase in its relative abundance has been found in a variety of diseases with liver function abnormalities after liver transplantation, nonalcoholic fatty liver, liver cancer, and other diseases. It was reported that the enrichment of Escherichia-*Shigella* is closely related to the production of endotoxin, which leads to endotoxemia, increased intestinal permeability, and liver damage (19). We found that hepatectomy increased the relative abundance of Escherichia-*Shigella*, and this might be the main reason for the observed inflammation. The F/B (*Firmicutes*/*Bacteroidetes*) value has been used as an intestinal evaluation index in the study of a variety of diseases, including alcoholic fatty liver, nonalcoholic fatty liver, liver oxidative stress injury, Alzheimer’s disease, and atherosclerosis (20). In this study, the ratio of F/B decreased at LH7, indicating a negative effect on the beagle’s gut after hepatectomy.

More importantly, we found that *Allobaculum* and *Turicibacter* abundance was negatively correlated with indicators of liver damage (ALT and AST) and proinflammatory factors (TNF-α, IL-1β, and IL-6) and positively correlated with anti-inflammatory factors (IL-4 and IL-10). In addition to our research, mounting evidence has suggested that intestinal microbiota are active in the gut-liver axis, and liver injury can change the microbial composition in the intestine, which is manifested as a lower diversity of intestinal microbes (21) that was consistent with the decrease in alpha diversity on LH7. Therefore, we conclude that the above three bacterial groups may be potential targets for improving the liver function and metabolism after liver surgery, and our findings clearly show that hepatectomy has a significant impact on the microbiota; further modulation of the gut microbiota may play an important role in recovery after hepatectomy.

Studies demonstrated that phenylalanine was primarily catabolized in the liver by phenylalanine hydroxylase (PAH) to produce tyrosine and that the metabolic control of phenylalanine concentrations in body fluids was critical for cognitive development and executive capacity (22). Our metabolomics results showed that both fecal and serum metabolomes were enriched in phenylalanine, tyrosine, and tryptophan biosynthesis and phenylalanine metabolism, which indicated that these pathways are important in liver recovery. As previously described by Lu et al. (23), kaempferol ameliorated the liver damage caused by nonalcoholic fatty liver disease (NAFLD) mainly through the regulation of the two pathways of phenylalanine, tyrosine and tryptophan biosynthesis and phenylalanine metabolism, and the metabolites they produce. Disturbances in the metabolism of phenylalanine and tyrosine can lead to the development of NAFLD, which is characterized primarily by liver damage.

Remarkably, our proteomics data showed significant downregulation of the phenylalanine metabolic pathway as a result of hepatectomy. In addition, aminoacyl-tRNA biosynthesis and riboflavin metabolism were significantly associated with recovery mechanisms after liver injury. It has been demonstrated that aspirin eugenol ester (AEE) protects against paraquat-induced acute liver injury (ALI) in rats by regulating metabolite production from phenylalanine, tyrosine, and tryptophan biosynthesis, aminoacyl-tRNA biosynthesis, phenylalanine metabolism, and other metabolic pathways (24). For example, fumaric acid, a metabolite of tyrosine, is involved in the tricarboxylic acid cycle, thereby affecting the body’s energy level. An imbalance between AMP and ATP can inactivate AMP-dependent protein kinase (AMPK), which leads to abnormal activation of apoptotic proteins and exacerbates liver damage. Thyroid hormone (TH) is a product of the phenylalanine/tyrosine pathway and can cause disturbances in hepatic lipid metabolism and further deterioration of liver function (25).

Interestingly, we showed specific upregulation of the serum metabolite l-phenylalanine, which is involved in three important signaling pathways: phenylalanine, tyrosine, and tryptophan biosynthesis, phenylalanine metabolism, and aminoacyl-tRNA biosynthesis. Further supporting evidence comes from a previous animal study (26) that showed ursodeoxycholic acid (UDCA), a by-product of intestinal bacterial metabolism, could mitigate liver dysfunction by significantly reducing l-phenylalanine and its downstream molecules and modulating the phenylalanine/tyrosine pathway. Similarly, another previous study revealed that microbial metabolites of aromatic amino acids such as phenylalanine, tyrosine, and especially phenylacetic acid were strongly associated with hepatic steatosis (27). As phenylalanine is a precursor of phenylacetic acid, increased phenylalanine levels could raise phenylacetic acid concentrations and promote hepatic disease (28). Huang et al. also demonstrated that a specific set of metabolites including phenylalanine, inosine, and bilirubin had great potential as biomarkers of drug-induced liver injury (DILI) for clinical diagnosis (29). Therefore, we concluded that phenylalanine, tyrosine, and tryptophan biosyntheses play important roles in the metabolic mechanism of liver injury. l-Phenylalanine could be a useful monitor and biomarker of the process of liver injury, and its regulation might be utilized to limit liver damage.

In the correlation analysis, we observed a significant association between the serum metabolite 3a,7a-dihydroxy-5b-cholestane, which was upregulated within 7 days after hepatectomy compared to preoperatively, and inflammatory factors and gut microbiota related to the liver injury. The KEGG results revealed that 3a,7a-dihydroxy-5b-cholestane enrichment was a component of the primary bile acid biosynthesis pathway. We speculate that primary bile acid biosynthesis could be an important metabolic pathway in the posthepatectomy response. Cholesterol is the main target of primary bile acids, and cholesterol, in turn, is an essential part of the energy generation process for recovery after hepatectomy. It was reported that primary bile acids were synthesized directly from cholesterol in hepatocytes but could be converted into secondary bile acids by gut microbes (30). Most bile acids are retained in the hepatic-intestinal circulation, and only a very small amount enters the blood circulation, but when the organism is affected by liver disease, the bile acid secretion function of the liver is impaired, which could account for the increase in related metabolites such as 3a,7a-dihydroxy-5b-cholestane in the serum metabolome during the 7-day postoperative period of liver injury. Researchers have shown that liver injuries such as NAFLD (31) and DILI (32) augment bile acid levels. The results of Jiao et al. (33), who studied serum bile acid levels and gut microbial composition in a rat model of NAFLD, suggested that future therapeutic interventions in the development of NAFLD could be achieved by regulating bile acid metabolism. A study by Ma et al. (34) reported that primary bile acid metabolism was closely linked to the development and prevention of liver tumors. Hence, primary bile acid biosynthesis is an important metabolic pathway to focus on during liver injury.

We also found that the metabolic pathway for aminoacyl-tRNA biosynthesis was enriched for fecal and serum metabolites at LH28 compared to LH0, including a significant increase in the fecal metabolite l-threonine and the serum metabolite l-arginine. Based on this finding, we hypothesized that aminoacyl-tRNA biosynthesis was an important metabolic pathway for liver recovery and that the upregulation of two metabolites, l-arginine and l-threonine, facilitated the recovery process after liver injury. Previous studies have reported that l-arginine was the most important metabolite in various metabolic models. l-Arginine is a major intermediate in the urea cycle and is the precursor of nitric oxide (NO), which has been implicated in various liver injuries (35). l-Arginine increased the activity of antioxidant enzymes and reduced malondialdehyde levels by promoting NO synthesis, thus mitigating tissue damage caused by oxidative stress (36). l-Threonine is an essential amino acid in mammalian cells and was shown to have beneficial effects on NAFLD by reducing the characteristic liver damage through synthesis of phospholipids and oxidation of fatty acids (37). Collectively, our findings support the conclusion that an increase in l-arginine and l-threonine levels is strongly associated with hepatic recovery and that manipulation of the biosynthesis of these signature target metabolites may be effective in promoting liver recovery after hepatectomy.

To further elucidate the mechanism of liver injury, we determined the proteomic profile response to hepatectomy at 7 days postoperation (postop). By analyzing the enrichment pathways of upregulated proteins, we found a significant increase in proteins associated with the mitogen-activated protein kinase (MAPK) signaling pathway. This pathway has been shown to regulate almost all hepatocyte physiological processes in previous studies, including the expression of genes involved in pretranscriptional, posttranscriptional, and translational processes. The MAPK pathway has been reported to be closely associated with liver injury and liver-related inflammatory responses, liver fibrosis, liver cancer, and other pathologies (38). For example, Ma et al. (39) showed that the MAPK pathway played a key role in the progression of nonalcoholic steatohepatitis. Ci et al. (40) confirmed that the MAPK signaling pathway was a potential molecular target for the treatment and prognostic evaluation of hepatocellular carcinoma. In a previous study, it was also found that blocking p38 MAPK promoted DNA replication during liver regeneration but maintained the hepatocyte cell cycle arrest in adult liver (41). Inhibition of the MAPK signaling pathway improved liver fibrosis (42), and blocking MAPK phosphorylation was anti-inflammatory and hepatoprotective (43). Moreover, it has also been reported that MAPK signaling plays an inhibitory role in the development of NAFLD (44). Further studies into the molecular mechanism of regulation of liver recovery and the role of the MAPK signaling pathway are required.

The protein-enrichment pathway analysis in the present study revealed that phenylalanine and tyrosine, and the tryptophan biosynthesis/phenylalanine metabolic pathways, which showed significant differences in the metabolome results, had the same downregulation trend in the proteomic profiling. We found that the peroxisome proliferator-activated receptor (PPAR)-mediated metabolic pathways that were demonstrated to be associated with liver injury in previous studies (45) showed a similar trend in this study. PPARs, which belong to the nuclear receptor hormone family, consist of three isoforms, PPARα, PPARβ, and PPARγ. Among these, PPARγ has been shown to be involved in hepatic ischemia-reperfusion injury. Treatment with a synthetic PPARγ ligand attenuated postischemic liver injury, while knockout of PPARγ exacerbated postischemic liver injury (46). Other studies have verified that the plant compound berberine may relieve fibrosis by regulating PPARγ and restoring lipid homeostasis via modulation of arachidonic acid metabolism (47). Further studies are needed to evaluate the effectiveness of PPARγ intervention by comparison with proteomic results. Taken together, we conclude that the PPAR signaling pathway is a key player in the process of liver injury and recovery, and future studies are warranted for experimental verification of our hypothesis.

### Conclusions.

In summary, our investigation integrates detailed clinical and multi-omics data to characterize the metabolic response to hepatectomy in beagle dogs. We also performed an interactional analysis to decipher the mechanism of liver injury and the body’s response. Our experiments identified the key molecules involved in the physiology of liver injury and recovery and also characterized the dysbiosis resulting from hepatectomy. As one outcome of the metabolomics and liver proteomics analyses, we determined that the fecal metabolite phenylacetylglycine and the serum metabolite l-phenylalanine could potentially be used as biomarkers for liver injury. Additionally, the fecal metabolite l-threonine and the serum metabolite l-arginine could potentially be used as key target metabolites for the liver recovery process. Moreover, the MAPK and PPAR signaling pathways significantly contributed to enhancing liver recovery. A limitation of this study is that further experiments are necessary to corroborate our findings in different species. This study does provide the foundation for future treatment of and recovery from common clinical diseases such as liver cancer and liver tumors and facilitates studies on the microbiota in the gut-liver axis.

## MATERIALS AND METHODS

### Experimental animals.

Eight healthy adult (7 to 8 months old, 7.1 ± 6 kg) female beagles were obtained from Beijing Marshall Biotechnology Co., Ltd., China (no. SYXK2019-0047). The experimental protocol was approved by the Animal Ethics Committee of Beijing University of Agriculture (BUAEC 2019-0205). Animal care and handling were performed in accordance with the *Guide for the Care and Use of Laboratory Animals* of the National Research Council (48). The dogs were housed individually and fed a standard canine diet (Beijing Keao Xieli Feed Co., Ltd., China) with *ad libitum* access to tap water. Animals were acclimated to their new environment for 14 days prior to their first fecal collection, during which time the health status of each animal was evaluated daily.

### Hepatectomy and sampling.

A purely laparoscopic left hemihepatectomy was performed under aseptic conditions on the dogs under general anesthesia as described in our previous study (49). Briefly, the left triangular ligament was cut first, the base of the left medial lobe was ligated with silk suture using a needle, and the liver parenchyma of the left medial lobe approximately 0.5 cm above the ligation site was removed with LigaSure (10 mm; Covidien Medical). The left lateral lobe was removed in the same manner. The resected lobes were placed in a sterile bag and then taken out through a minilaparotomy port that was created by extending the trocar site. After ensuring hemostasis, the laparoscopic equipment was removed and the ports were sutured. All surgical procedures were performed under aseptic conditions by the same team of a surgeon and two assistants. Buprenorphine, 0.01 mg/kg of body weight intramuscularly (i.m.) (Hansen Pharma Co., Ltd., Changsha, China), was administered every 8 h for 3 days after surgery for analgesia. All animals were clinically examined daily until they recovered to normal preoperative state.

In the morning before surgery (LH0) and after successful modeling by laparoscopic hepatectomy (LH) on days 7 (LH7) and 28 (LH28), we collected fecal samples and stored them in liquid nitrogen for 16S rRNA gene sequencing and metabolomics analysis. Blood samples were collected from the forelimb veins before surgery and after the surgery on days 1, 3, 7, 14, and 28, allowed to clot, and centrifuged for 10 min at 3,000 × *g*. The serum was aliquoted into two microcentrifuge tubes and immediately stored at −80°C for later measurement of clinical indicators. Blood samples were taken at LH0, LH7, and LH28 for serum metabolomic analysis. We took appropriate amounts of liver tissue before (LH0) and on day 7 after (LH7) surgery and stored the specimens at −70°C for subsequent proteomic analysis.

### Serum and hepatic biochemical indicators.

Serum tumor necrosis factor alpha (TNF-α), interleukin-1β (IL-1β), interleukin-6 (IL-6), interleukin-4 (IL-4), and interleukin-10 (IL-10) were measured using enzyme-linked immunosorbent assay kits (Huaying Biological Technology Co., Ltd., Beijing, China).

The levels of serum aspartate transferase (AST) and alanine transaminase (ALT) were determined with a biochemical analyzer (Mindray Biomedical Electronics Co., Ltd., Shenzhen, China) and a liver function test kit (Yapu Biological Technology Co., Ltd., Shanghai, China). The intra- and interassay coefficients of variation were 8.76 and 4.46%, respectively.

### 16S rRNA gene sequencing and analysis.

Total DNA extraction and PCR amplification were performed as previously described (50, 51). The sequences of the primers were as follows: 338F, 5′-ACTCCTACGGGAGGCAGCAG-3′; 806R, 5′-GGACTACHVGGGTWTCTAAT-3′.

PCR products were purified using the AxyPrep DNA gel extraction kit (Axygen Biosciences, Union City, CA, USA), and paired-end sequencing was performed using an Illumina MiSeq Platform (Illumina, San Diego, CA, USA) according to standard protocols by Majorbio Bio-Pharm Technology Co. Ltd. (Shanghai, China) (50).

For bioinformatics analysis of microbial sequencing results, raw fastq files were demultiplexed, quality filtered by Trimmomatic (52), and merged by FLASH with the reported criteria (51). Operational taxonomic units (OTUs) were clustered with a 97% similarity cutoff (50) using UPARSE (version 7.1, http://drive5.com/uparse/). Chimeric sequences were identified and removed using UCHIME. The taxonomy of the acquired OTUs was analyzed using the Ribosomal Database Project (RDP) classifier algorithm (http://rdp.cme.msu.edu/) against the SILVA (release 123, http://www.arb-silva.de) (53) 16S rRNA gene database with a confidence threshold of 70%. Alpha diversity analyses, including community richness parameters (Chao and Sobs) and community diversity parameters (Shannon and Simpson), were calculated using the Mothur software (50). Principal-coordinate analysis (PCoA) was performed, with unweighted UniFrac as distance measure, to evaluate beta diversity. Bacterial taxonomic distributions of sample communities were visualized using the R package software. Linear discriminant analysis (LDA) coupled with effect size (LEfSe) (54) measurements (based on nonparametric factorial Kruskal-Wallis rank sum test and the Wilcoxon rank sum test) was used to identify significantly different taxa (biomarkers) between groups (LH0 and LH7), with a *P* value of <0.01 and an LDA score threshold of 4 (55). Correlations between clinical parameter indices and 16S rRNA gene sequence data are presented in the form of a heatmap diagram based on Spearman’s correlation coefficient. At the genus level, the correlation coefficient of the *R* value was color mapped onto the gut microbiota, showing the correlation of each gut species with the clinical indices. *R* values are presented in different colors, and the color card shows the color partition of different *R* values (*P* < 0.05, *; *P* < 0.01, **) (56).

### Metabolomics.

For extracting fecal metabolites, we added 25 mg of each fecal sample to 500 μL of acetonitrile-methanol-water (2:2:1, vol/vol/vol) containing an isotopically labeled internal standard mixture. After vortexing for 30 s, the samples were homogenized at 35 Hz for 4 min and sonicated for 5 min, three times, in an ice-water bath. The samples were then held for 1 h at −40°C and centrifuged at 12,000 × *g* for 15 min at 4°C. The supernatants (400 μL) were transferred to EP tubes and dried under vacuum and then dissolved in 200 μL of 50% acetonitrile, vortexed for 30 s, sonicated for 10 min on ice, and centrifuged at 13,000 × *g* for 15 min at 4°C. Afterward, 75-μL aliquots of supernatant were taken for liquid chromatography with tandem mass spectrometry analysis (LC-MS/MS). An equal aliquot of each supernatant (10 μL) was used as the quality control (QC) sample. Likewise, for the serum samples, we pipetted 50-μL aliquots into a 1.5-mL tube, added 200 μL of methanol-acetonitrile (1:1, vol/vol) containing an isotopically labeled internal standard mixture, vortexed and mixed the tubes for 30 s, sonicated the tubes for 10 min on ice, and held the tubes at −40°C for 1 h. The samples were then centrifuged at 12,000 × *g* for 15 min at 4°C, and the supernatants were collected. The samples were analyzed using a Vanquish ultrahigh-pressure liquid chromatography (UHPLC) system (Thermo Fisher Scientific) in conjunction with a Thermo Q Exactive HFX mass spectrometer.

The raw metabolomics data were converted into mzXML format with ProteoWizard and then processed using a procedure developed with R for automatic data analysis for peak detection, extraction, alignment, and integration (Biotree, Shanghai, China). Individual peaks were filtered to remove noise based on their relative standard deviation. Peak area data with vacancies of ≤50% in a single group or null values of ≤50% in all groups were retained. Missing raw data points were filled in with a minimum value of one-half, and internal standards were used for data standardization and normalization. Afterward, the database was searched for all metabolites, and they were sorted according to type. Multivariate analyses including principal-component analysis (PCA) and orthogonal correction partial least-squares discriminant analysis (OPLS-DA) were conducted using SIMCA software (V15.0.2; Sartorius Stedim Data Analytics AB, Umea, Sweden). The OPLS-DA was used to filter out orthogonal variables in metabolites that were not correlated with categorical variables, thus ensuring that only accurately annotated metabolites were selected for differential analysis. Metabolites differentially expressed between two treatments were identified based on variable importance in projection (VIP) from OPLS-DA and statistical analysis (VIP of >1 and *P* of <0.05), and further hierarchical cluster analysis (HCA) and heatmap construction were performed for the different metabolites (57). After mapping the differentially produced metabolites to authoritative metabolite databases such as KEGG and PubChem and obtaining matches for the differential metabolites, we searched the pathway database for Homo sapiens and performed metabolic pathway analysis to determine the enrichment pathways corresponding to the different metabolites.

### Proteomics.

Protein extraction was performed on eight beagle hepatectomy samples pooled before and 7 days after surgery. To remove residual blood cells, the pooled liver samples were rinsed twice with ice-cold phosphate-buffered saline (PBS, pH 7.4) and then transferred into radioimmunoprecipitation assay (RIPA) lysis buffer (Thermo Fisher Scientific, MA, USA) containing protease inhibitors at a final concentration of 1% (vol/vol). Liver tissues were homogenized and then sonicated for 5 times and 10 s using an ultrasonic instrument (Sonics & Materials, Inc., Newtown, CT, USA). Homogenates were kept at 4°C for 40 min and then centrifuged at 12,000 × *g* for 10 min at 4°C. Protein concentration was determined by bicinchoninic acid assay (Thermo Fisher Scientific, MA, USA), and samples were prepared for proteomic analysis by Tandem Mass Tag (TMT) labeling (58).

Aliquots of the desalted peptides (5 μg) were injected for nanoflow LC-MS/MS analysis using an LTQ Orbitrap Velos mass spectrometer (Thermo Fisher Scientific, Waltham, MA, USA). Technical duplication was conducted. The iTRAQ project was used to compare the proteins between the two groups in terms of upregulated proteins (FC of >1.2) and downregulated proteins (FC of <0.83) (*P* < 0.05). The identified differential proteins were then screened with GO and KEGG databases to obtain comprehensive functional information about the annotated proteins. The KEGG pathway enrichment analysis was also performed on the proteins in the protein set according to the Majorbio Cloud Platform development process, using the same principle as the GO functional enrichment analysis.

### Gut microbiome-metabolome correlation analysis.

Spearman correlation analysis was used to analyze the association between selected analytes and other data sets (metabolomics and microbiota). The correlations between clinical parameters (TNF-α, IL-6, IL-4, IL-1β, and IL-10) and bacterial/serum metabolites were assessed in the LH0 and LH7 groups, respectively, as well as for associations between the top 10 bacterial species and the top 15 differential metabolites, which were screened to show significant differences. The results were visualized as heatmap plots. *R* values indicate the correlation between each metabolite and the genus-level gut microbiota as different colors. The correlation coefficient and *P* value for each species-metabolite pair were calculated and considered significantly associated with a cutoff *P* value of <0.05. This was performed using the Corr.test function in the stats package of R language tools.

### Statistical analysis.

Values were expressed as mean ± standard deviation (SD) for continuous variables. Gut microbial domains, phyla, and genera were compared using the Wilcoxon rank sum test, with an adjusted false-discovery rate (FDR) of *P* < 0.05 being considered significantly different. Student’s *t* test (two-sided) was used to compare the differences for other multi-omics analyses between preoperative and postoperative time points. Differential protein expression was screened according to fold change (FC of >1.2 or FC of <0.83), using Fisher’s exact test (*P* < 0.05) for pathway enrichment analysis with all proteins as background.

### Data availability.

Due to the confidentiality of data, the data which support the findings of this study are available only in a redacted form upon request. All raw sequence data were uploaded to NCBI (PRJNA844202).

## References

[1] Rahbari NN, Birgin E, Bork U, Mehrabi A, Reißfelder C, Weitz J. 2021. Anterior approach vs conventional hepatectomy for resection of colorectal liver metastasis. JAMA Surg 156:31–40. doi:10.1001/jamasurg.2020.5050. 33147332 PMC7643041

[2] Sahay P, Jain K, Sinha P, Das B, Mishra A, Kesarwani A, Sahu P, Mohan KV, Kumar M, Nagarajan P, Upadhyay P. 2019. Generation of a rat model of acute liver failure by combining 70% partial hepatectomy and acetaminophen. J Vis Exp (153). doi:10.3791/60146. 31840655

[3] Nithyananthan S, Thirunavukkarasu C. 2019. Chemotherapeutic doses of arsenic trioxide delays hepatic regeneration by oxidative stress and hepatocyte apoptosis in partial hepatectomy rat. Toxicol Appl Pharmacol 382:114760. doi:10.1016/j.taap.2019.114760. 31525389

[4] Kajiura D, Yamanaka-Okumura H, Hirayama A, Tatano H, Endo K, Honma M, Igarashi K, Shoji F, Ikeda S, Yamaguchi N, Katayama T, Morine Y, Imura S, Utsunomiya T, Soga T, Tomita M, Shimada M. 2018. Perioperative serum and urine metabolome analyses in patients with hepatocellular carcinoma undergoing partial hepatectomy. Nutrition 58:110–119. doi:10.1016/j.nut.2018.06.002. 30391689

[5] Albillos A, de Gottardi A, Rescigno M. 2020. The gut-liver axis in liver disease: pathophysiological basis for therapy. J Hepatol 72:558–577. doi:10.1016/j.jhep.2019.10.003. 31622696

[6] Liu HX, Rocha CS, Dandekar S, Wan YJ. 2016. Functional analysis of the relationship between intestinal microbiota and the expression of hepatic genes and pathways during the course of liver regeneration. J Hepatol 64:641–650. doi:10.1016/j.jhep.2015.09.022. 26453969 PMC4761311

[7] Hagymási K, Bacsárdi A, Egresi A, Berta E, Tulassay Z, Lengyel G. 2018. The role of gut microbiota in chronic liver diseases, and treatment possibilities. Orv Hetil 159:1465–1474. (In Hungarian.) . doi:10.1556/650.2018.31178. 30175608

[8] Brandl K, Schnabl B. 2017. Intestinal microbiota and nonalcoholic steatohepatitis. Curr Opin Gastroenterol 33:128–133. doi:10.1097/MOG.0000000000000349. 28257306 PMC5662009

[9] Cornide-Petronio ME, Álvarez-Mercado AI, Jiménez-Castro MB, Peralta C. 2020. Current knowledge about the effect of nutritional status, supplemented nutrition diet, and gut microbiota on hepatic ischemia-reperfusion and regeneration in liver surgery. Nutrients 12:284. doi:10.3390/nu12020284. 31973190 PMC7071361

[10] Wu Y. 2016. Alleviation of chronic alcoholic liver injury by Lactobacillus rhamnosus through adjustment of normal intestinal flora. Master’s thesis. Jilin Agricultural University, Changchun, China.

[11] Franzosa EA, Hsu T, Sirota-Madi A, Shafquat A, Abu-Ali G, Morgan XC, Huttenhower C. 2015. Sequencing and beyond: integrating molecular ‘omics’ for microbial community profiling. Nat Rev Microbiol 13:360–372. doi:10.1038/nrmicro3451. 25915636 PMC4800835

[12] Fan ST, Mau Lo C, Poon RTP, Yeung C, Leung Liu C, Yuen WK, Ming Lam C, Ng KKC, Chan SC. 2011. Continuous improvement of survival outcomes of resection of hepatocellular carcinoma: a 20-year experience. Ann Surg 253:745–758. doi:10.1097/SLA.0b013e3182111195. 21475015

[13] Honda G, Kurata M, Okuda Y, Kobayashi S, Tadano S, Yamaguchi T, Matsumoto H, Nakano D, Takahashi K. 2013. Totally laparoscopic hepatectomy exposing the major vessels. J Hepatobiliary Pancreat Sci 20:435–440. doi:10.1007/s00534-012-0586-7. 23269462

[14] Troisi RI, Van Huysse J, Berrevoet F, Vandenbossche B, Sainz-Barriga M, Vinci A, Ricciardi S, Bocchetti T, Rogiers X, de Hemptinne B. 2011. Evolution of laparoscopic left lateral sectionectomy without the Pringle maneuver: through resection of benign and malignant tumors to living liver donation. Surg Endosc 25:79–87. doi:10.1007/s00464-010-1133-8. 20532569 PMC3003798

[15] Aldrighetti L, Pulitanò C, Arru M, Catena M, Guzzetti E, Casati M, Ferla G. 2008. Ultrasonic-mediated laparoscopic liver transection. Am J Surg 195:270–272. doi:10.1016/j.amjsurg.2007.02.022. 18154765

[16] Gayet B, Cavaliere D, Vibert E, Perniceni T, Levard H, Denet C, Christidis C, Blain A, Mal F. 2007. Totally laparoscopic right hepatectomy. Am J Surg 194:685–689. doi:10.1016/j.amjsurg.2006.11.044. 17936436

[17] Liu Y, Wang H, Zhang J, Shao J, Shi J. 2010. Effects of different carbon dioxide pneumoperitoneum on the circulatory system of dogs. J Northeast Agric Univ 41:113–116.

[18] Velázquez KT, Enos RT, Bader JE, Sougiannis AT, Carson MS, Chatzistamou I, Carson JA, Nagarkatti PS, Nagarkatti M, Murphy EA. 2019. Prolonged high-fat-diet feeding promotes non-alcoholic fatty liver disease and alters gut microbiota in mice. World J Hepatol 11:619–637. doi:10.4254/wjh.v11.i8.619. 31528245 PMC6717713

[19] Shen F, Zheng RD, Sun XQ, Ding WJ, Wang XY, Fan JG. 2017. Gut microbiota dysbiosis in patients with non-alcoholic fatty liver disease. Hepatobiliary Pancreat Dis Int 16:375–381. doi:10.1016/S1499-3872(17)60019-5. 28823367

[20] Li X, Wang Y, Xing Y, Xing R, Liu Y, Xu Y. 2019. Changes of gut microbiota during silybin-mediated treatment of high-fat diet-induced non-alcoholic fatty liver disease in mice. Hepatol Res 50:5–14. doi:10.1111/hepr.13444. 31661720

[21] Lu HF, Ren ZG, Li A, Zhang H, Xu SY, Jiang JW, Zhou L, Ling Q, Wang BH, Cui GY, Chen XH, Zheng SS, Li LJ. 2019. Fecal microbiome data distinguish liver recipients with normal and abnormal liver function from healthy controls. Front Microbiol 10:1518. doi:10.3389/fmicb.2019.01518. 31333622 PMC6619441

[22] Eichinger A, Danecka MK, Möglich T, Borsch J, Woidy M, Büttner L, Muntau AC, Gersting SW. 2018. Secondary BH4 deficiency links protein homeostasis to regulation of phenylalanine metabolism. Hum Mol Genet 27:1732–1742. doi:10.1093/hmg/ddy079. 29514280

[23] Lu Y, Shao M, Xiang H, Zheng P, Wu T, Ji G. 2020. Integrative transcriptomics and metabolomics explore the mechanism of kaempferol on improving nonalcoholic steatohepatitis. Food Funct 11:10058–10069. doi:10.1039/d0fo02123g. 33135718

[24] Zhang ZD, Yang YJ, Liu XW, Qin Z, Li SH, Li JY. 2020. The protective effect of aspirin eugenol ester on paraquat-induced acute liver injury rats. Front Med 7:589011. doi:10.3389/fmed.2020.589011. PMC7773779 33392217

[25] Zhao P, Sun X, Chaggan C, Liao Z, In Wong K, He F, Singh S, Loomba R, Karin M, Witztum JL, Saltiel AR. 2020. An AMPK-caspase-6 axis controls liver damage in nonalcoholic steatohepatitis. Science 367:652–660. doi:10.1126/science.aay0542. 32029622 PMC8012106

[26] Kim DJ, Yoon S, Ji SC, Yang J, Kim YK, Lee S, Yu KS, Jang IJ, Chung JY, Cho JY. 2018. Author correction: ursodeoxycholic acid improves liver function via phenylalanine/tyrosine pathway and microbiome remodelling in patients with liver dysfunction. Sci Rep 9:17003. doi:10.1038/s41598-019-53737-7. PMC6851095 31719650

[27] Hoyles L, Fernández-Real JM, Federici M, Serino M, Abbott J, Charpentier J, Heymes C, Luque JL, Anthony E, Barton RH, Chilloux J, Myridakis A, Martinez-Gili L, Moreno-Navarrete JM, Benhamed F, Azalbert V, Blasco-Baque V, Puig J, Xifra G, Ricart W, Tomlinson C, Woodbridge M, Cardellini M, Davato F, Cardolini I, Porzio O, Gentileschi P, Lopez F, Foufelle F, Butcher SA, Holmes E, Nicholson JK, Postic C, Burcelin R, Dumas ME. 2018. Molecular phenomics and metagenomics of hepatic steatosis in non-diabetic obese women. Nat Med 24:1070–1080. doi:10.1038/s41591-018-0061-3. 29942096 PMC6140997

[28] Liu Y, Luo Y, Wang X, Luo L, Sun K, Zeng L. 2020. Gut microbiome and metabolome response of pu-erh tea on metabolism disorder induced by chronic alcohol consumption. J Agric Food Chem 68:6615–6627. doi:10.1021/acs.jafc.0c01947. 32419453

[29] Huang Y, Zhao X, Zhang ZT, Chen SS, Li SS, Shi Z, Jing J, Huang A, Guo YM, Bai ZF, Zou ZS, Xiao XH, Wang JB, Niu M. 2020. Metabolomics profiling and diagnosis biomarkers searching for drug-induced liver injury implicated to *Polygonum multiflorum*: a cross-sectional cohort study. Front Med 7:592434. doi:10.3389/fmed.2020.592434. PMC7734208 33330552

[30] Luo Y, Gao F, Chang R, Zhang X, Zhong J, Wen J, Wu J, Zhou T. 2021. Metabolomics based comprehensive investigation of Gardeniae Fructus induced hepatotoxicity. Food Chem Toxicol 153:112250. doi:10.1016/j.fct.2021.112250. 33964367

[31] Lake AD, Novak P, Shipkova P, Aranibar N, Robertson D, Reily MD, Lu Z, Lehman-McKeeman LD, Cherrington NJ. 2013. Decreased hepatotoxic bile acid composition and altered synthesis in progressive human nonalcoholic fatty liver disease. Toxicol Appl Pharmacol 268:132–140. doi:10.1016/j.taap.2013.01.022. 23391614 PMC3627549

[32] Slopianka M, Herrmann A, Pavkovic M, Ellinger-Ziegelbauer H, Ernst R, Mally A, Keck M, Riefke B. 2017. Quantitative targeted bile acid profiling as new markers for DILI in a model of methapyrilene-induced liver injury in rats. Toxicology 386:1–10. doi:10.1016/j.tox.2017.05.009. 28529062

[33] Jiao N, Baker SS, Chapa-Rodriguez A, Liu W, Nugent CA, Tsompana M, Mastrandrea L, Buck MJ, Baker RD, Genco RJ, Zhu R, Zhu L. 2018. Suppressed hepatic bile acid signalling despite elevated production of primary and secondary bile acids in NAFLD. Gut 67:1881–1891. doi:10.1136/gutjnl-2017-314307. 28774887

[34] Ma C, Han M, Heinrich B, Fu Q, Zhang Q, Sandhu M, Agdashian D, Terabe M, Berzofsky JA, Fako V, Ritz T, Longerich T, Theriot CM, McCulloch JA, Roy S, Yuan W, Thovarai V, Sen SK, Ruchirawat M, Korangy F, Wang XW, Trinchieri G, Greten TF. 2018. Gut microbiome-mediated bile acid metabolism regulates liver cancer via NKT cells. Science 360:eaan5931. doi:10.1126/science.aan5931. 29798856 PMC6407885

[35] Morris CR, Hamilton-Reeves J, Martindale RG, Sarav M, Ochoa Gautier JB. 2017. Acquired amino acid deficiencies: a focus on arginine and glutamine. Nutr Clin Pract 32(1_suppl):30S–47S. doi:10.1177/0884533617691250. 28388380

[36] Liu Y, Paterson M, Baumgardt SL, Irwin MG, Xia Z, Bosnjak ZJ, Ge ZD. 2019. Vascular endothelial growth factor regulation of endothelial nitric oxide synthase phosphorylation is involved in isoflurane cardiac preconditioning. Cardiovasc Res 115:168–178. doi:10.1093/cvr/cvy157. 29931049 PMC6302266

[37] Zhang Z, Zhou Y, Lin Y, Li Y, Xia B, Lin L, Liao D. 2020. GC-MS-based metabolomics research on the anti-hyperlipidaemic activity of Prunella vulgaris L. polysaccharides. Int J Biol Macromol 159:461–473. doi:10.1016/j.ijbiomac.2020.05.003. 32387363

[38] Gui WF, Xu S, Dang ZS, Zhao YM. 2019. In vitro and in vivo effect of MAPK signal transduction pathway inhibitors on Echinococcus multilocularis. J Parasitol 105:146–154. doi:10.1645/18-121. 30807708

[39] Ma JM, Gao LL, Zhang MW, Gao QH, Tao XJ, FAN YN, YANG JJ. 2021. Role and mechanism of *Lycium barbarum* polysaccharide combined with aerobic exercise in improving nonalcoholic steatohepatitis in rats. J Clin Hepatol 37:1–6. doi:10.16050/j.cnki.issn1674-6309.2021.05.002.

[40] Ci D, Lin K, Lu Z, Zhao X, Wang X. 2016. The role of MAPK signaling pathway in development/progression and treatment of liver cancer. J Clin Hepatol 32:1810–1813.

[41] Campbell JS, Argast GM, Yuen SY, Hayes B, Fausto N. 2010. Inactivation of p38 MAPK during liver regeneration. Int J Biochem Cell Biol 43:180–188. doi:10.1016/j.biocel.2010.08.002. 20708092 PMC3022113

[42] Shen X, Guo H, Xu J, Wang J. 2019. Inhibition of lncRNA HULC improves hepatic fibrosis and hepatocyte apoptosis by inhibiting the MAPK signaling pathway in rats with nonalcoholic fatty liver disease. J Cell Physiol 234:18169–18179. doi:10.1002/jcp.28450. 30908654

[43] Jiang J, Yan L, Shi Z, Wang L, Shan L, Efferth T. 2019. Hepatoprotective and anti-inflammatory effects of total flavonoids of Qu Zhi Ke (peel of Citrus changshan-huyou) on non-alcoholic fatty liver disease in rats via modulation of NF-κB and MAPKs. Phytomedicine 64:153082. doi:10.1016/j.phymed.2019.153082. 31541796

[44] Wang Y, Zeng Z, Guan L, Ao R. 2020. GRHL2 induces liver fibrosis and intestinal mucosal barrier dysfunction in non-alcoholic fatty liver disease via microRNA-200 and the MAPK pathway. J Cell Mol Med 24:6107–6119. doi:10.1111/jcmm.15212. 32324317 PMC7294114

[45] Berthier A, Johanns M, Zummo FP, Lefebvre P, Staels B. 2021. PPARs in liver physiology. Biochim Biophys Acta Mol Basis Dis 1867:166097. doi:10.1016/j.bbadis.2021.166097. 33524529

[46] Kuboki S, Shin T, Huber N, Eismann T, Galloway E, Schuster R, Blanchard J, Zingarelli B, Lentsch AB. 2007. Peroxisome proliferator-activated receptor-gamma protects against hepatic ischemia/reperfusion injury in mice. Hepatology (Baltimore, Md) 47:215–224. doi:10.1002/hep.21963. 18085707

[47] Liu X, Wang L, Tan S, Chen Z, Wu B, Wu X. 2022. Therapeutic effects of berberine on liver fibrosis are associated with lipid metabolism and intestinal flora. Front Pharmacol 13:814871. doi:10.3389/fphar.2022.814871. 35308208 PMC8924518

[48] National Research Council. 2011. Guide for the care and use of laboratory animals, 8th ed. National Academies Press, Washington, DC.

[49] Zhang H, Wang J, Cao Y, Zhang ZN, Shen J, Tong J, Wang HB. 2020. Laparoscopic left hemihepatectomy in small dogs: an easy and effective new technique. Acta Vet Brno 89:367–373. doi:10.2754/avb202089040367.

[50] Tong J, Zhang H, Zhang Y, Xiong B, Jiang L. 2019. Microbiome and metabolome analyses of milk from dairy cows with subclinical *Streptococcus agalactiae* mastitis-potential biomarkers. Front Microbiol 10:2547. doi:10.3389/fmicb.2019.02547. 31781063 PMC6851174

[51] Zheng Y, Hao X, Lin X, Zheng Q, Zhang W, Zhou P, Li S. 2018. Bacterial diversity in the feces of dogs with CPV infection. Microb Pathog 121:70–76. doi:10.1016/j.micpath.2018.04.043. 29709688

[52] Bolger AM, Lohse M, Usadel B. 2014. Trimmomatic: a flexible trimmer for Illumina sequence data. Bioinformatics 30:2114–2120. doi:10.1093/bioinformatics/btu170. 24695404 PMC4103590

[53] Quast C, Pruesse E, Yilmaz P, Gerken J, Schweer T, Yarza P, Peplies J, Glöckner FO. 2013. The SILVA ribosomal RNA gene database project: improved data processing and web-based tools. Nucleic Acids Res 41:D590–D596. doi:10.1093/nar/gks1219. 23193283 PMC3531112

[54] Hou K, Tong J, Zhang H, Gao S, Guo Y, Niu H, Xiong B, Jiang L. 2020. Microbiome and metabolic changes in milk in response to artemisinin supplementation in dairy cows. AMB Express 10:154. doi:10.1186/s13568-020-01080-w. 32833065 PMC7445214

[55] Zhu L, Zhang D, Zhu H, Zhu J, Weng S, Dong L, Liu T, Hu Y, Shen X. 2018. Berberine treatment increases Akkermansia in the gut and improves high-fat diet-induced atherosclerosis in Apoe^−/−^ mice. Atherosclerosis 268:117–126. doi:10.1016/j.atherosclerosis.2017.11.023. 29202334

[56] Zhang X, Wu Q, Zhao Y, Yang X. 2019. *Decaisnea insignis* seed oil inhibits trimethylamine-*N*-oxide formation and remodels intestinal microbiota to alleviate liver dysfunction in l-carnitine feeding mice. J Agric Food Chem 67:13082–13092. doi:10.1021/acs.jafc.9b05383. 31671940

[57] Wang Y, Nan X, Zhao Y, Jiang L, Wang H, Zhang F, Hua D, Liu J, Yao J, Yang L, Luo Q, Xiong B. 2021. Dietary supplementation of inulin ameliorates subclinical mastitis via regulation of rumen microbial community and metabolites in dairy cows. Microbiol Spectr 9:e0010521. doi:10.1128/Spectrum.00105-21. 34494854 PMC8557905

[58] Gao Y, Lee H, Kwon OK, Cheng Z, Tan M, Kim KT, Lee S. 2019. Profiling of histidine phosphoproteome in Danio rerio by TiO_2_ enrichment. Proteomics 19:e1800471. doi:10.1002/pmic.201800471. 30864180

