# Supplementary material for: Mechanism of Metabolic Response to Hepatectomy by Integrated Analysis of Gut Microbiota, Metabolomics, and Proteomics
Source: Microbiol Spectr. 2023 Apr 10;11(3):e02067-22. doi: 10.1128/spectrum.02067-22 (PMC10269556; doi:10.1128/spectrum.02067-22)
Supplement: Supplemental file 1 — Supplemental material. Download spectrum.02067-22-s0001.pdf, PDF file, 0.2 MB [file spectrum.02067-22-s0001.pdf]

Supplementary Table S1

Name	mz	rt	MEAN LH7	MEAN LH0	VIP	P-VALUE	Q-VALUE	FOLD CHANGE	LOG FC
7-Oxocholesterol	401.34	33.62	0.78	1.97	2.02	0.0001	0.05	0.40	-1.34
D-Galactarate	210.04	41.95	0.10	0.01	1.95	0.0275	0.15	8.11	3.02
D-Biotin	262.13	378.10	0.43	0.23	1.94	0.0006	0.07	1.89	0.92
Coniine	255.28	137.54	0.03	0.05	1.93	0.0019	0.07	0.62	-0.68
Acetyl Tyrosine Ethyl Ester	274.10	310.76	0.44	0.15	1.89	0.0018	0.07	2.90	1.54
Myristoleic acid	244.23	44.27	0.46	1.11	1.87	0.0025	0.08	0.41	-1.28
3-Hydroxymandelic acid	151.04	130.08	0.06	0.10	1.86	0.0026	0.08	0.66	-0.59
Bufexamac	206.12	237.63	0.31	1.29	1.81	0.0327	0.16	0.24	-2.04
Citramalic acid	149.04	173.53	0.06	0.09	1.80	0.0016	0.07	0.66	-0.59
beta-Estradiol	237.16	242.23	1.03	13.37	1.80	0.0317	0.16	0.08	-3.70
Betaine aldehyde	162.11	287.66	0.52	1.49	1.78	0.0043	0.09	0.35	-1.52
2-Hydroxyadenine	152.06	214.23	0.29	0.09	1.77	0.0126	0.13	3.36	1.75
Palmitoylethanolamide	299.27	167.62	0.02	0.05	1.75	0.0022	0.08	0.51	-0.98
Indole	118.06	224.95	0.15	0.18	1.07	0.0160	0.14	0.85	-0.24
Phenylacetylglycine	235.11	73.73	0.25	1.70	1.70	0.0397	0.17	0.15	-2.74

Note: Top 15 fecal metabolites screened by OPLS-DA model with VIP >1 and $p < 0.05$ were significantly different on LH7 compared to LH0.

Supplementary Table S2

Name	mz	rt	MEAN LH28	MEAN LH0	VIP	P-VALUE	Q-VALUE	FOLD CHANGE	LOG FC
4-Pyridinecarboxylic acid	184.06	335.28	0.74	0.43	2.53	0.0007	0.22	1.71	0.78
Arg-Cys	242.11	138.84	0.52	0.31	2.45	0.0021	0.23	1.67	0.74
DL-Norvaline	257.15	338.94	0.88	0.60	2.43	0.0032	0.24	1.46	0.55
2'-O-methylcytidine	258.11	181.49	0.65	0.28	2.43	0.0136	0.27	2.31	1.21
Acetyl-DL-Valine	142.08	93.92	0.38	0.20	2.41	0.0017	0.23	1.89	0.92
Val-Asp	250.14	241.99	0.08	0.41	2.40	0.0141	0.27	0.20	-2.30
Ajmalicine	419.13	36.63	0.08	0.15	2.36	0.0003	0.22	0.55	-0.87
Formylanthranilic acid	226.07	289.46	14.97	10.38	2.27	0.0063	0.24	1.44	0.53
Dimethylbenzimidazole	147.09	186.66	0.12	0.08	2.24	0.0083	0.25	1.50	0.58
D-Erythrose 4-phosphate	242.04	289.48	0.44	0.31	2.24	0.0090	0.25	1.42	0.51
Lys-Arg	347.18	440.90	0.30	0.15	2.13	0.0177	0.28	2.02	1.01
Riboflavin	377.15	199.88	0.22	0.45	1.40	0.0451	0.31	0.49	-1.03
L-Threonine	142.05	289.45	0.41	0.29	1.97	0.0284	0.29	1.40	0.49
3-Hydroxyanthranilic acid	154.05	328.54	3.28	2.26	2.12	0.0089	0.25	1.45	0.53
L-Pyroglutamic acid	276.12	373.41	0.98	0.57	2.07	0.0346	0.30	1.73	0.79

Note: Top 15 fecal metabolites screened by OPLS-DA model with VIP >1 and p < 0.05 were significantly different on LH28 compared to LH0.

Supplementary Table S3

Name	rt	mz	MEAN LH7	MEAN LH0	VIP	P-VALUE	Q-VALUE	FOLD CHANGE	LOG FC
3a,7a-Dihydroxy-5b-cholestane	23.66	405.37	0.01	0.00	2.38	0.0004	0.17	2.77	1.47
PS(20:5(5Z,8Z,11Z,14Z,17Z)/18:3(6Z,9Z,12Z))	240.65	804.49	0.12	0.26	2.38	0.0041	0.17	0.45	-1.17
Morpholine	206.43	88.08	0.43	2.06	2.28	0.0086	0.17	0.21	-2.25
3-Aminobutanoic acid	318.91	104.07	2.19	1.34	1.81	0.0170	0.19	1.63	0.71
L-Phenylalanine	278.43	166.09	9.71	7.56	2.19	0.0060	0.17	1.28	0.36
Tetrahydroaldosterone-3-glucuronide	93.86	541.26	0.19	0.08	2.18	0.0368	0.27	2.46	1.30
beta-Elemenone	33.32	219.17	0.03	0.91	2.17	0.0185	0.20	0.04	-4.72
8-Butanoylneosolaniol	79.36	453.21	0.31	0.13	2.16	0.0382	0.27	2.36	1.24
Erythrabyssin II	74.16	393.21	0.42	0.18	2.15	0.0421	0.28	2.31	1.20
Kanzonol I	79.44	437.23	0.41	0.18	2.15	0.0407	0.28	2.31	1.21
Citronellyl beta-sophoroside	85.24	481.26	0.27	0.09	2.10	0.0040	0.17	3.01	1.59
3-Methylhistidine	395.83	170.09	3.60	2.15	2.01	0.0057	0.17	1.67	0.74
beta-Solamarine	150.90	868.51	0.03	0.08	2.01	0.0105	0.17	0.44	-1.19
Proline betaine	291.25	144.10	1.00	1.63	1.53	0.0438	0.29	0.61	-0.71

Isopentyl mercaptan	318.89	105.07	0.07	0.04	1.92	0.0091	0.17	2.09	1.06

Note: Top 15 serum metabolites screened by OPLS-DA model with VIP >1 and $p < 0.05$ were significantly different on LH7 compared to LH0.

Supplementary Table S4

Name	rt	mz	MEAN LH28	MEAN LH0	VIP	P-VALUE	Q-VALUE	FOLD CHANGE	LOG FC
1H-Indole-3-carboxaldehyde	48.34	146.06	0.07	0.05	2.52	0.0002	0.08	1.44	0.52
2,3,4,5,6,7-Hexahydro-7-methylcyclopent[b]azepin-8(1H)-one	33.41	166.12	0.03	0.11	2.47	0.0003	0.08	0.25	-2.00
beta-Sinensal	191.40	219.17	0.01	0.05	2.45	0.0050	0.11	0.26	-1.95
Isovalerylglucuronide	325.39	279.11	0.02	0.02	2.44	0.0018	0.09	1.29	0.36
1-Methylnicotinamide	310.52	137.07	1.72	0.70	2.41	0.0009	0.08	2.47	1.31
Niacinamide	59.76	123.06	0.39	0.17	1.73	0.0493	0.31	2.24	1.16
4-Amino-2-methyl-1-naphthol	25.44	174.09	0.04	0.09	2.28	0.0016	0.09	0.44	-1.17
Threoninyl-Proline	411.72	217.12	0.01	0.01	2.25	0.0365	0.27	1.89	0.92
PC(22:4(7Z,10Z,13Z,16Z)/P-18:0)	156.30	822.63	0.84	0.66	2.11	0.0013	0.09	1.26	0.34
L-Octanoylcarnitine	228.77	288.22	0.03	0.05	2.10	0.0036	0.10	0.64	-0.64
3-Aminobutanoic acid	318.91	104.07	2.11	1.34	1.72	0.0404	0.28	1.58	0.66
L-Arginine	524.12	175.12	5.44	4.54	2.02	0.0202	0.21	1.20	0.26
Riboflavin	235.12	377.15	0.02	0.01	2.06	0.0191	0.20	1.48	0.57
5-Hydroxy-L-tryptophan	50.83	221.09	0.12	0.07	2.05	0.0110	0.15	1.66	0.73
1,7-Dimethylguanosine	209.71	312.13	0.02	0.01	2.04	0.0123	0.16	1.46	0.54

Note: Top 15 serum metabolites screened by OPLS-DA model with VIP >1 and $p <$

0.05 were significantly different on LH28 compared to LH0.

Supplementary Table S5

	TNF_α	IL_1β	IL_6	IL_10	IL_4
3a,7a-Dihydroxy-5b-cholestane	0.66	0.89	0.61	-0.75	-0.44
PS(20:5(5Z,8Z,11Z,14Z,17Z)/18:3(6Z,9Z,12Z))	-0.54	-0.78	-0.74	0.68	0.49
Morpholine	-0.57	-0.71	-0.49	0.56	0.35
3−Aminobutanoic acid	0.61	0.59	0.38	-0.64	-0.66
L−Phenylalanine	0.52	0.73	0.48	-0.65	-0.42
Tetrahydroaldosterone-3-glucuronide	0.43	0.75	0.40	-0.72	-0.29
beta-Elemenone	-0.57	-0.72	-0.48	0.40	0.18
8-Butanoylneosolaniol	0.32	0.57	0.33	-0.57	-0.31
Erythrabyssin II	0.39	0.70	0.46	-0.62	-0.19
Kanzonol I	0.36	0.70	0.38	-0.66	-0.25
Citronellyl beta-sophoroside	0.58	0.62	0.61	-0.72	-0.52
3−Methylhistidine	-0.14	-0.37	-0.08	-0.04	-0.24
beta-Solamarine	-0.54	-0.60	-0.68	0.42	0.28
Proline betaine	-0.10	-0.55	-0.28	0.43	0.16
Isopentyl mercaptan	0.62	0.61	0.46	-0.70	-0.67

Note: Comparing LH0 with LH7,correlation coefficient values for the five main inflammatory indicators and the top fifteen fecal differential metabolites.

Supplementary Table S6

	Peptoclostridium	Collinsella	Blautia	Escherichia-Shigella	Romboutsia	Turicibacter	Allobaculum	Lactobacillus	Erysipelotrichaceae	Lachnospiraceae
2−Hydroxyadenine	-0.21	0.13	-0.34	0.52	0.31	-0.57	-0.50	0.01	-0.48	-0.44
D−Biotin	-0.36	-0.03	-0.43	0.81	0.49	-0.52	-0.65	-0.34	-0.73	-0.65
Acetyl Tyrosine Ethyl Ester	-0.27	-0.04	-0.51	0.80	0.42	-0.53	-0.46	-0.36	-0.57	-0.68
D−Galactarate	-0.30	-0.09	-0.47	0.79	0.15	-0.52	-0.46	-0.36	-0.46	-0.74
Riboflavin	0.06	-0.04	-0.22	0.01	0.29	-0.16	-0.22	-0.24	-0.41	-0.17
Citramalic acid	0.16	-0.11	-0.05	-0.50	-0.10	0.13	0.23	-0.05	0.18	0.53
7−Oxocholesterol	0.45	-0.02	0.06	-0.55	-0.31	0.29	0.40	-0.08	0.06	0.51
3−Hydroxyanthranilic acid	0.17	-0.06	0.10	-0.16	-0.33	0.09	0.20	0.00	0.54	0.34
Indole	0.30	0.31	0.41	-0.51	0.12	0.43	0.14	-0.14	0.20	0.15
Betaine aldehyde	0.36	0.09	0.26	-0.66	-0.16	0.41	0.46	0.02	0.52	0.56
4−Pyridinecarboxylic acid	-0.28	-0.18	-0.56	0.39	0.41	-0.34	-0.25	-0.31	-0.15	-0.17
L−Threonine	0.26	0.34	0.01	-0.16	-0.45	0.16	0.23	-0.23	0.41	0.21
Phenylacetylglycine	-0.10	-0.24	0.09	-0.42	-0.31	0.16	0.21	-0.13	0.41	0.56
3−Hydroxymandelic acid	0.12	-0.38	0.14	-0.59	-0.27	0.34	0.53	0.14	0.44	0.54
1H−Indole−3−propanoic acid	0.55	0.08	0.24	-0.61	-0.17	0.28	0.43	0.32	0.38	0.55

Note: Comparing LH0 with LH7,correlation coefficient values for the top ten most abundant genera and the top fifteen fecal differential metabolites.

Supplementary Table S7

	Peptoclostridium	Collinsella	Blautia	Escherichia-Shigella	Romboutsia	Turicibacter	Allobaculum	Lactobacillus	Erysipelotrichaceae	Lachnospiraceae
3a,7a-Dihydroxy-5b-cholestane	-0.37	-0.02	-0.05	0.68	0.15	-0.38	-0.56	0.04	-0.25	-0.55
PS(20:5(5Z,8Z,11Z,14Z,17Z)/18:3(6Z,9Z,12Z))	0.05	-0.11	0.16	-0.62	-0.36	0.24	0.40	0.20	0.52	0.66
Morpholine	0.28	-0.36	-0.06	-0.64	-0.42	0.19	0.50	-0.07	0.44	0.58
3−Aminobutanoic acid	-0.24	0.07	-0.23	0.49	0.31	-0.28	-0.24	0.25	-0.37	-0.42
L−Phenylalanine	-0.31	0.31	0.01	0.47	0.63	-0.19	-0.37	0.26	-0.33	-0.43
Tetrahydroaldosterone-3-glucuronide	-0.36	-0.13	0.11	0.49	0.59	-0.18	-0.50	0.00	-0.18	-0.35
beta-Elemenone	0.34	-0.26	-0.04	-0.60	-0.25	0.29	0.66	0.01	0.37	0.51
8-Butanoylneosolaniol	-0.27	0.05	-0.04	0.44	0.79	-0.23	-0.48	-0.06	-0.21	-0.37
Erythrabyssin II	-0.34	-0.26	-0.10	0.58	0.55	-0.35	-0.60	-0.13	-0.21	-0.29
Kanzonol I	-0.38	-0.09	0.02	0.45	0.70	-0.27	-0.52	0.06	-0.15	-0.32
Citronellyl beta-sophoroside	-0.26	-0.14	-0.46	0.76	0.67	-0.56	-0.56	-0.25	-0.61	-0.55
3−Methylhistidine	0.42	-0.21	-0.21	-0.20	0.00	0.01	0.38	-0.04	-0.29	0.11
beta-Solamarine	0.07	-0.26	0.20	-0.57	-0.34	0.26	0.42	0.21	0.53	0.55
Proline betaine	-0.01	-0.27	-0.06	-0.18	-0.51	-0.14	0.03	-0.12	0.27	0.34
Isopentyl mercaptan	-0.23	0.06	-0.29	0.54	0.34	-0.34	-0.22	0.32	-0.42	-0.42

Note: Comparing LH0 with LH7,correlation coefficient values for the top ten most abundant genera and the top fifteen serum differential metabolites.

Supplementary Table S8

Accession	FC(LH7/LH0)	Log2FC(LH7/LH0)	P_value(LH7/LH0)	Regulate
A0A5F4D168	2.79	1.48	0.0024	up
A0A5F4CS47	2.74	1.45	0.0004	up
E2RBA1	1.82	0.86	0.0468	up
E2RE67	2.08	1.06	0.0289	up
E2RM48	2.29	1.2	0.0009	up
A0A5F4CCV9	2.16	1.11	0.0015	up
J9P673	2.58	1.37	0.0021	up
H9GWG4	1.68	0.75	0.0036	up
Q2TLZ1	2.85	1.51	0.0113	up
C7C419	2.2	1.14	0.0252	up
P99504	1.75	0.81	0.0127	up
E2REC1	1.7	0.77	0.0189	up
A0A5F4CRD6	2.1	1.07	0.0186	up
E2RK33	1.55	0.63	0.0228	up
H9GWY1	1.46	0.55	0.0178	up
F6UME0	1.38	0.46	0.0331	up
A0A5F4DJA2	2.12	1.08	0.0164	up
A0A5F4CNP2	1.74	0.8	0.0309	up
J9P185	1.57	0.65	0.0231	up
E2QXJ4	1.7	0.77	0.0208	up
F1Q2A2	2.36	1.24	0	up
A0A5F4D0B9	1.43	0.51	0.0076	up
A0A5F4BST4	1.52	0.6	0.0303	up

J9NVP2	1.88	0.91	0.0003	up
A0A5F4CRG8	1.54	0.62	0.0102	up
F1PCR2	1.67	0.74	0.0025	up
E2R1X9	1.92	0.94	0.0461	up
J9P2K0	1.9	0.93	0.0201	up
A0A5F4CAX4	2	1	0.0284	up
A0A5F4CYU6	1.76	0.82	0.0117	up
F1PVG0	1.8	0.85	0.0349	up
F1Q2B9	1.43	0.51	0.0201	up
F1Q054	1.39	0.48	0.0222	up
E2RBS1	1.73	0.79	0.0419	up
A0A5F4C0R5	2.51	1.33	0.0144	up
A0A5F4BSD0	1.83	0.87	0.0259	up
A0A5F4CPX4	1.47	0.56	0.0038	up
J9JHQ3	2.01	1.01	0.0487	up
A0A5F4C4C3	1.31	0.39	0.0283	up
A0A5F4DDD7	1.74	0.8	0.031	up
E2RFD8	1.81	0.86	0.0252	up
A0A5F4BZ89	1.48	0.57	0.0413	up
F6Y4A3	1.62	0.69	0.0318	up
J9NSE6	2.13	1.09	0.0179	up
A0A5F4CNQ4	1.6	0.68	0.0354	up
A0A5F4D9V3	1.63	0.71	0.0198	up
J9NS24	1.9	0.92	0.0222	up
J9P017	1.44	0.52	0.0313	up
F1PKI4	1.75	0.8	0.0126	up

E2QWZ9	1.6	0.68	0.0436	up
E2RM54	1.71	0.77	0.0454	up
F1PB37	1.33	0.41	0.0423	up
A0A5F4C4Q1	1.89	0.92	0.0029	up
F6VA50	1.6	0.68	0.011	up
E2R8E3	1.46	0.55	0.001	up
A0A5F4CZM1	1.38	0.47	0.041	up
A0A5F4C646	1.66	0.73	0.0333	up
F1PEI2	1.59	0.67	0.0229	up
F1PU47	1.29	0.37	0.0215	up
E2RJV6	1.42	0.51	0.0194	up
J9PAQ1	1.52	0.61	0.0002	up
E2RLB8	1.37	0.45	0.0467	up
F1PVC0	1.61	0.68	0.0391	up
Q5TJE4	1.4	0.49	0.0397	up
A0A5F4CGS5	1.38	0.46	0.0043	up
J9NW84	1.55	0.63	0.048	up
J9NYK7	1.5	0.59	0.0162	up
E2R906	1.57	0.65	0.0452	up
A0A5F4BQT8	1.32	0.4	0.034	up
A0A5F4DF23	1.56	0.64	0.0218	up
F1PZ83	1.31	0.39	0.0192	up
F1PXG4	1.69	0.75	0.0152	up
A0A5F4CS65	1.35	0.44	0.0362	up
P60058	1.51	0.6	0.0279	up
A0A3B0ITT8	1.34	0.43	0.0486	up

E2RKB0	1.49	0.58	0.0301	up
E2RLN5	1.28	0.35	0.0239	up
F1PQM2	1.23	0.3	0.0407	up
A0A5F4CXI9	1.58	0.66	0.0424	up
F6XN94	2.19	1.13	0.0031	up
J9P5I6	1.73	0.79	0.0355	up
E2RIA1	1.47	0.56	0.0123	up
A0A5F4D0M8	1.32	0.4	0.0308	up
A0A5F4CTS2	1.94	0.96	0.0208	up
E2QWP1	1.22	0.29	0.0315	up
A0A5F4DC35	1.28	0.35	0.0431	up
E2RQE3	1.43	0.51	0.0311	up
J9NSI1	1.46	0.55	0.0334	up
A0A5F4DFJ9	1.34	0.42	0.0368	up
F6UQ33	1.45	0.54	0.0413	up
O78115	1.96	0.97	0.0482	up
E2RSK1	1.82	0.86	0.0335	up
A0A5F4BSC2	1.52	0.61	0.0398	up
E2R8S6	1.39	0.48	0.0353	up
J9P6U1	1.49	0.58	0.0358	up
A0A5F4CIC4	1.44	0.53	0.0347	up
A0A5F4CP46	1.48	0.57	0.0474	up
A0A5F4CTD0	1.29	0.37	0.0281	up
F1PQK7	1.41	0.5	0.0266	up
F1Q4B4	1.37	0.45	0.005	up
J9P366	1.25	0.33	0.0413	up

F1PGT1	1.3	0.37	0.0393	up
J9NX46	1.66	0.73	0.0203	up
F1PSB4	1.37	0.46	0.0248	up
F1Q3Y2	1.51	0.59	0.0242	up
E2RFL0	1.7	0.77	0.007	up
E2REN0	1.32	0.4	0.0178	up
E2RTL4	1.32	0.4	0.0223	up
J9JHH5	1.47	0.55	0.0104	up
Q3ZKN1	1.68	0.75	0.0313	up
E2QUW7	1.77	0.82	0.0124	up
A0A5F4D645	1.36	0.44	0.0194	up
E2RAW0	1.46	0.54	0.042	up
X5IHJ5	1.54	0.62	0.0469	up
F6V9R9	1.41	0.49	0.0302	up
F1Q4I3	1.3	0.37	0.0364	up
A0A5F4DB79	1.38	0.47	0.0358	up
E2RH99	1.34	0.42	0.0475	up
E2R479	1.39	0.47	0.0443	up
J9P969	1.4	0.49	0.0101	up
A0A5F4BVL8	1.58	0.66	0.0415	up
J9P316	1.28	0.35	0.0235	up
A0A5F4C9J4	1.26	0.33	0.0444	up
E2RQF8	1.49	0.57	0.0185	up
A0A5F4BS96	1.34	0.42	0.0484	up
E2RB77	1.57	0.65	0.0311	up
F1Q2H2	1.4	0.48	0.0416	up

A0A5F4CJ16	1.41	0.49	0.0235	up
E2RT43	1.48	0.57	0.0447	up
E2RSH4	1.45	0.54	0.0377	up
A0A5F4DGH1	1.49	0.57	0.0408	up
E2RFM5	1.34	0.42	0.0249	up
E2R0F7	1.35	0.43	0.0275	up
A0A5F4C461	1.41	0.49	0.0447	up
E2R5Q1	1.37	0.45	0.0453	up
E2REU6	1.27	0.34	0.013	up
E2QTI7	1.42	0.5	0.0261	up
F1PG16	1.44	0.53	0.0258	up
F1PJY1	2.01	1.01	0.0361	up
F6UQZ9	1.35	0.43	0.0157	up
J9P3S0	1.39	0.48	0.0022	up
A0A5F4CCI6	1.35	0.43	0.0329	up
A0A5F4DDG2	1.46	0.55	0.0088	up
E2R913	1.4	0.49	0.0498	up
A0A5F4D3V6	1.37	0.45	0.0365	up
F1PNG2	1.24	0.31	0.0342	up
J9P2F1	1.33	0.41	0.0059	up
A0A5F4C1Z1	1.23	0.29	0.0075	up
A0A5F4CYB3	1.37	0.45	0.0017	up
E2RB37	1.33	0.42	0.0241	up
F1PHP1	1.32	0.4	0.0048	up
A0A0A0MPC0	1.36	0.44	0.0339	up
F1PP40	1.44	0.53	0.0269	up

E2RCF9	1.24	0.31	0.0151	up
A0A5F4D3B4	1.22	0.29	0.0458	up
E2RAW7	1.86	0.9	0.0439	up
J9P393	1.41	0.49	0.0206	up
E2R292	1.47	0.56	0.0448	up
E2RSU1	1.42	0.51	0.0323	up
A0A5F4BYR3	1.88	0.91	0.0111	up
F1PA71	1.72	0.79	0.0491	up
F1PAG7	1.29	0.37	0.0208	up
E2QT63	1.43	0.51	0.0339	up
A0A5F4C9H9	1.33	0.41	0.0178	up
J9NZA2	1.26	0.33	0.0331	up
F1P914	1.42	0.51	0.0054	up
A0A5F4BR32	1.34	0.42	0.0446	up
F1PIL4	1.3	0.37	0.0323	up
J9P2W5	1.77	0.83	0.0426	up
A0A5F4CKJ7	1.52	0.6	0.035	up
E2RRN8	1.28	0.36	0.0316	up
E2R0D6	1.33	0.42	0.0015	up
E2QSE3	1.3	0.38	0.0488	up
E2RPM1	1.6	0.68	0.0462	up
A0A5F4CJY2	1.57	0.65	0.0173	up
F1PB95	1.27	0.34	0.0406	up
J9NSQ4	1.42	0.5	0.0185	up
E2RKJ6	1.33	0.41	0.0354	up
E2RB00	1.38	0.47	0.0396	up

F1PRT9	1.33	0.41	0.0023	up
E2R5Z5	1.21	0.28	0.0464	up
A0A5F4CHH4	1.29	0.36	0.0376	up
E2RIF9	1.68	0.75	0.0349	up
J9P2U5	1.24	0.31	0.0435	up
A0A5F4C0Z8	1.28	0.36	0.0493	up
A0A5F4BSE6	1.25	0.32	0.0382	up
F1P9U4	1.36	0.44	0.0003	up
E2RNW5	1.31	0.38	0.0043	up
A0A5F4CTP0	1.21	0.27	0.009	up
F1PRL5	1.37	0.46	0.0302	up
J9NT64	1.59	0.67	0.0229	up
F1PPT2	1.31	0.39	0.0002	up
E2RSI0	1.37	0.45	0.0384	up
E2R2G5	1.39	0.47	0.0471	up
F1Q1F5	2.01	1.01	0.0093	up
E2RB43	1.41	0.5	0.0058	up
J9NZX6	1.23	0.3	0.0079	up
A0A5F4C9Z1	1.43	0.51	0.0272	up
E2RP67	1.4	0.48	0.0362	up
F1Q018	1.41	0.5	0.0089	up
F1PPM7	1.2	0.27	0.0219	up
A0A5F4BS08	1.43	0.52	0.0347	up
A0A5F4C873	1.67	0.74	0.0394	up
E2RGP2	1.25	0.32	0.0162	up
F6XRM5	1.48	0.56	0.0065	up

A0A5F4DC98	1.23	0.3	0.0466	up
E2R5S7	1.21	0.28	0.0302	up
E2RR40	1.39	0.48	0.0419	up
F6XJP3	1.43	0.51	0.0424	up
F1P6W7	1.29	0.37	0.0293	up
F1P9Z4	1.39	0.48	0.0378	up
E2R8A7	1.28	0.35	0.0074	up
A0A5F4D0H1	1.24	0.31	0.0368	up
E2QSF4	1.26	0.34	0.0168	up
E2R9J8	1.25	0.33	0.0234	up
A0A5F4D8F9	1.32	0.4	0.0376	up
E2R8P1	1.47	0.55	0.0038	up
F1P8Z5	1.27	0.35	0.0002	up
F1PFB3	1.24	0.31	0.0313	up
A0A5F4CJE6	0.54	-0.88	0.0373	down
A0A5F4CD04	1.27	0.35	0.0185	up
F1PUY0	1.25	0.32	0.0014	up
E2RJM1	1.27	0.35	0.0115	up
A0A5F4C566	1.39	0.47	0.0141	up
A0A5F4CJ48	1.24	0.32	0.0496	up
A0A5F4CNX4	1.33	0.42	0.0229	up
E2R1D2	1.31	0.39	0.0496	up
E2R6F8	1.26	0.33	0.0378	up
E2RAS3	1.8	0.85	0.0493	up
J9P9R6	1.43	0.51	0.0167	up
E2RCJ3	1.24	0.32	0.0122	up

A0A5F4CGD6	1.4	0.49	0.0056	up
E2R7N4	1.25	0.32	0.0232	up
A0A5F4CAY2	1.32	0.4	0.0047	up
F1PXG3	1.36	0.44	0.0205	up
F1P8R0	1.47	0.55	0.006	up
F1PCT1	1.28	0.36	0.0147	up
F1PPJ4	1.31	0.39	0.007	up
E2RPA3	1.29	0.37	0.0497	up
E2QWX5	1.28	0.36	0.033	up
A0A5F4C6Y2	1.26	0.33	0.0104	up
F1PT17	1.26	0.33	0.0208	up
E2RL06	1.47	0.56	0.0293	up
E2RHI0	1.27	0.35	0.0303	up
F1PLD0	1.73	0.79	0.0377	up
A0A5F4D553	1.28	0.36	0.0458	up
J9P024	1.4	0.49	0.0441	up
E2RK29	1.34	0.43	0.0276	up
F1PMG8	1.23	0.3	0.0251	up
A0A5F4CX70	1.27	0.34	0.0085	up
E2RMA0	1.24	0.31	0.048	up
A0A5F4CWP7	1.29	0.37	0.0362	up
J9PB32	1.26	0.33	0.0121	up
E2QU31	1.26	0.34	0.0241	up
E2RES1	1.34	0.42	0.0091	up
A0A5F4CWJ4	1.22	0.29	0.0486	up
E2R9E3	1.27	0.34	0.0352	up

F1PPF7	1.43	0.51	0.0403	up
J9P9T3	1.28	0.36	0.0021	up
A0A5F4DNH3	1.38	0.46	0.0363	up
A0A5F4BWK5	1.28	0.36	0.0379	up
J9NVJ3	1.25	0.33	0.0381	up
A0A5F4C069	1.37	0.45	0.0354	up
F1PE94	1.26	0.33	0.0195	up
E2QVF9	1.23	0.3	0.0091	up
A0A5F4CHN0	1.27	0.35	0.045	up
A0A5F4D9P4	1.37	0.46	0.0027	up
Q6QNF4	1.31	0.39	0.0476	up
A0A5F4D9C2	1.4	0.49	0.0303	up
E2QY31	1.24	0.31	0.0088	up
A0A5F4DHU8	1.45	0.53	0.0141	up
F1PYS6	1.55	0.63	0.0444	up
F1PB46	1.35	0.44	0.0482	up
E2RK67	1.22	0.28	0.0052	up
A0A5F4C0K3	1.23	0.3	0.0168	up
F1PSZ2	1.28	0.36	0.0442	up
F1P6Z3	1.26	0.33	0.0143	up
F6XAE9	1.36	0.45	0.0251	up
E2QVV4	1.39	0.47	0.0375	up
F1PQV4	1.23	0.3	0.0132	up
E2QUV3	0.54	-0.88	0.0128	down
F1Q284	1.23	0.29	0.0254	up
F1PAD0	1.22	0.28	0.0109	up

F1PLN1	1.25	0.32	0.033	up
E2QTL0	1.3	0.38	0.0426	up
E2RFF0	1.2	0.27	0.0365	up
J9NXM2	1.3	0.38	0.0102	up
P28490	1.36	0.44	0.0413	up
E2RDI0	1.25	0.32	0.0162	up
J9P6E2	1.26	0.33	0.0445	up
F1PA10	1.21	0.28	0.007	up
E1B2G6	1.45	0.54	0.0297	up
E2QY42	1.23	0.3	0.012	up
F1PTE2	1.22	0.28	0.0167	up
F1PTA5	1.37	0.45	0.0243	up
A0A5F4BRK2	1.31	0.39	0.0215	up
A0A5F4C5X8	1.22	0.29	0.0321	up
P35521	1.3	0.38	0.0339	up
F1PLP6	1.34	0.42	0.0266	up
A0A5F4DFV1	1.39	0.48	0.0436	up
J9NSN4	1.69	0.76	0.0386	up
E2R4R2	1.23	0.29	0.0041	up
E2R5G6	1.23	0.3	0.0441	up
F6X7F9	1.29	0.37	0.0466	up
J9PB88	1.43	0.52	0.0454	up
E2R3X7	1.26	0.33	0.0129	up
F1Q255	1.22	0.29	0.0363	up
A0A5F4CD83	1.42	0.51	0.0404	up
E2R7L1	1.28	0.36	0.0087	up

A0A5F4D8U4	1.35	0.44	0.0243	up
J9P817	1.55	0.63	0.0438	up
A0A5F4BZZ0	1.22	0.28	0.0182	up
J9P6U5	1.27	0.35	0.0058	up
A0A5F4CUS0	1.29	0.37	0.0382	up
J9NRF6	1.25	0.32	0.0054	up
J9NSU1	1.21	0.28	0.0396	up
E2RME9	1.28	0.35	0.0379	up
E2RCU6	1.28	0.36	0.0113	up
A0A5F4BQ87	0.82	-0.28	0.044	down
J9NRY3	1.22	0.29	0.0006	up
J9NXS1	1.27	0.34	0.0191	up
F1P8G4	1.43	0.52	0.0345	up
A0A5F4CKZ9	1.56	0.64	0.0301	up
F1PZA0	0.83	-0.28	0.0222	down
E2RQS7	1.31	0.39	0.0084	up
F1Q1E1	0.78	-0.37	0.0076	down
E2RJE5	1.24	0.31	0.0186	up
E2RSW1	1.56	0.64	0.0196	up
F1PDJ5	0.66	-0.59	0.0175	down
A0A5F4D9W8	1.26	0.33	0.0082	up
J9NYQ3	1.68	0.74	0.036	up
F6V5W3	1.3	0.38	0.0344	up
F1PWZ5	1.43	0.52	0.0247	up
J9NSY4	1.35	0.44	0.0161	up
Q307K7	1.7	0.76	0.0179	up

C4MSN9	0.68	-0.56	0.0297	down
A0A0B4J196	1.48	0.57	0.0262	up
P58027	1.2	0.26	0.0316	up
E2RR38	0.78	-0.36	0.0371	down
J9NT73	0.73	-0.45	0.028	down
A0A5F4CST1	0.69	-0.55	0.0378	down
E2RFB0	0.61	-0.71	0.0113	down
A0A5F4CYH8	0.82	-0.28	0.0069	down
A0A5F4D358	0.75	-0.42	0.0318	down
A0A5F4CJZ5	1.28	0.36	0.0227	up
E2QUU5	0.81	-0.3	0.0388	down
C4MSD6	0.68	-0.55	0.0497	down
E2RAT9	1.31	0.39	0.0442	up
F1PIP0	0.81	-0.31	0.0129	down
J9NZ97	1.24	0.31	0.0449	up
J9P2R5	0.81	-0.3	0.0243	down
A0A5F4CQQ7	1.41	0.5	0.0311	up
J9P028	0.83	-0.28	0.0204	down
E2QVU9	0.8	-0.32	0.0093	down
A0A0B4J194	0.78	-0.35	0.0311	down
A0A5F4CMY8	0.68	-0.55	0.0148	down
F1P6W6	0.8	-0.32	0.0191	down
E2QYD2	0.82	-0.29	0.0291	down
J9P260	0.77	-0.37	0.0016	down
B3RFJ1	0.79	-0.34	0.0362	down
F1PLP2	0.78	-0.36	0.0445	down

A0A5F4C529	1.9	0.92	0.046	up
J9P6J9	0.8	-0.32	0.0235	down
A0A5F4D9D0	0.79	-0.34	0.0052	down
A0A5F4CHX9	0.81	-0.3	0.0002	down
E2RF28	0.81	-0.31	0.0151	down
F1PGS8	1.31	0.39	0.0413	up
A0A5F4DDJ1	0.82	-0.28	0.014	down
A0A5F4CKZ4	0.82	-0.28	0.0184	down
E2RTH5	0.72	-0.47	0.003	down
E2RNX1	0.51	-0.98	0.0262	down
A0A5F4DIW0	0.81	-0.3	0.0323	down
A0A5F4CQ69	0.81	-0.31	0.0404	down
A0A5F4D0E1	0.79	-0.34	0.0014	down
E2RK86	0.79	-0.33	0.0131	down
A0A5F4DEK7	0.74	-0.44	0.0074	down
J9NRH5	0.69	-0.53	0.0196	down
J9P4N5	0.7	-0.51	0.0046	down
J9P7P2	0.78	-0.37	0.0156	down
F1P6A6	0.77	-0.38	0.0015	down
A0A5F4D1G5	0.69	-0.53	0.0321	down
F1PST4	0.73	-0.46	0.0408	down
E2RLY6	0.73	-0.46	0.0199	down
E2RR27	0.77	-0.37	0.0032	down
J9NUF1	0.71	-0.5	0.0106	down
F6XHT8	0.68	-0.56	0.008	down
A0A5F4CIP3	0.63	-0.66	0.0031	down

E2RQ22	0.74	-0.42	0.0044	down
F1PN69	0.81	-0.3	0.0451	down
A0A5F4CN68	0.77	-0.37	0.0135	down
E2R375	0.6	-0.74	0.0187	down
F1PB77	0.81	-0.31	0.0339	down
E2RNV4	0.69	-0.55	0.0061	down
E2QRT4	0.6	-0.73	0.0267	down
J9NU67	0.67	-0.58	0.003	down
A0A5F4DA85	0.77	-0.38	0.0379	down
J9NSE8	0.74	-0.44	0.0285	down
A0A5F4CUN6	0.75	-0.41	0.0304	down
P42929	0.75	-0.42	0.0026	down
A0A5F4BRM0	0.55	-0.85	0.0151	down
P49819	0.69	-0.54	0.0256	down
E2RAC3	0.76	-0.4	0.0265	down
A0A172RTP4	0.51	-0.98	0.0081	down
E2RHL4	0.72	-0.47	0.0255	down
E2RTB5	0.59	-0.77	0.0054	down
A0A5F4C4C9	0.55	-0.86	0.0158	down
E2RPM0	0.57	-0.81	0.0013	down
J9P276	0.71	-0.49	0.026	down
A0A5F4CRX9	0.66	-0.59	0.0339	down
A0A5F4CZ48	0.69	-0.54	0.0329	down
A0A5F4D8A3	0.64	-0.65	0.0499	down
A0A5F4BUL8	0.59	-0.76	0.0189	down

Note: Comparing LH0 with LH7, a total of 412 proteins were significantly differentially expressed (FC>1.2 or <0.83，P<0.05).
